# Young children’s screen time during the first COVID-19 lockdown in 12 countries

**DOI:** 10.1038/s41598-022-05840-5

**Published:** 2022-02-07

**Authors:** Christina Bergmann, Nevena Dimitrova, Khadeejah Alaslani, Alaa Almohammadi, Haifa Alroqi, Suzanne Aussems, Mihaela Barokova, Catherine Davies, Nayeli Gonzalez-Gomez, Shannon P. Gibson, Naomi Havron, Tzipi Horowitz-Kraus, Junko Kanero, Natalia Kartushina, Christina Keller, Julien Mayor, Roger Mundry, Jeanne Shinskey, Nivedita Mani

**Affiliations:** 1 Max Planck Institute for Psycholinguistics, Nijmegen, The Netherlands; 2 Faculty of Social Work of Lausanne, University of Applied Sciences and Arts of Western Switzerland (HES-SO), Lausanne, Switzerland; 3 Purdue University, West Lafayette, USA; 4 King Abdulaziz University, Jeddah, Saudi Arabia; 5 University of Warwick, Coventry, UK; 6 Boston University, Boston, USA; 7 University of Leeds, Leeds, UK; 8 Centre for Psychological Research, Oxford Brookes University, Oxford, UK; 9 University of Haifa, Haifa, Israel; 10 Educational Neuroimaging Center, Technion-Israel Institute of Technology, Haifa, Israel; 11 Sabanicı University, Istanbul, Turkey; 12 University of Oslo, Oslo, Norway; 13 University of Göttingen, Goßlerstr. 14, 37073 Göttingen, Germany; 14 Cognitive Ethology Laboratory, German Primate Center, Göttingen, Germany; 15 Department for Primate Cognition, Georg-August-University Goettingen, Göttingen, Germany; 16 Leibniz ScienceCampus Primate Cognition, Göttingen, Germany; 17 Royal Holloway University of London, London, UK

**Keywords:** Psychology, Human behaviour

## Abstract

Older children with online schooling requirements, unsurprisingly, were reported to have increased screen time during the first COVID-19 lockdown in many countries. Here, we ask whether younger children with no similar online schooling requirements also had increased screen time during lockdown. We examined children’s screen time during the first COVID-19 lockdown in a large cohort (n = 2209) of 8-to-36-month-olds sampled from 15 labs across 12 countries. Caregivers reported that toddlers with no online schooling requirements were exposed to more screen time during lockdown than before lockdown. While this was exacerbated for countries with longer lockdowns, there was no evidence that the increase in screen time during lockdown was associated with socio-demographic variables, such as child age and socio-economic status (SES). However, screen time *during* lockdown was negatively associated with SES and positively associated with child age, caregiver screen time, and attitudes towards children’s screen time. The results highlight the impact of the COVID-19 lockdown on young children’s screen time.

In March 2020, the COVID-19 outbreak led to lockdowns and social distancing measures around the globe: Social interactions were restricted and many parents had to work from home while taking care of their children, because childcare facilities and schools were (partially) closed. The move to online schooling led school-aged children to spend many hours a day in front of screens, interacting with their teachers and classmates. Unsurprisingly then, older children (3- to 17-year-olds) in many countries (Australia, China, France, Germany, Italy, Netherlands, South Korea, Spain, UK, USA; see Table 1) spent extended periods engaged with digital media during lockdown. What about the youngest members of our society, toddlers and pre-schoolers, who did not attend online schooling? What was their experience with screen time during this lockdown period? And what were the factors that shaped their screen time exposure during lockdown? Were they exposed to increased periods of screen time during lockdown as their caregivers battled to keep them occupied while working from home, and was there an association between potential increases in screen time during lockdown and children’s language development? The current study addresses these questions by examining screen time during lockdown in 8–36 month-olds using data from 15 labs across 12 countries (Canada, France, Germany, Israel, Norway, Poland, Russia, Saudi Arabia, Switzerland, Turkey, UK, USA).

**Table 1:** Previous findings on lockdown-related increases to children’s screen time.

Country	Age	Screen time effect
Canada	5- to 11-years	95% of children not meeting guidelines for physical activity due to sedentary behaviour including screen time^1^
China	6- to 17-years	30 h more screen time per week^2^
France	6- to 10-years	62% of children had increased screen time^3^
Germany	4- to 17-years	One hour more screen time per day^4^
Italy	6- to 18-years	Almost 5 h more screen time per day in children with obesity^5^
Netherlands	6- to 14-years	Self-reported screen time increased by 59–62 min per day^6^
South Korea	age not reported	81% of children had increased screen time^7^
Spain	8- to 16-years	2 h more screen time per day^8^
USA	< 18 years	‘Dramatic’ increase in screen time^9^
Multi-country	3- to 7-years	50 min more screen time per day^10^

## Factors associated with screen time in early childhood

Children start using digital media devices early in life^11–13^, with US data suggesting a 32% increase in children’s screen time over the last two decades^14^. Children’s screen time also increases with age^11,13,15–18^ with two-year-olds in the US having less than an hour of daily screen time and two- to four-year-olds having on average 2.5 h of daily screen time^13^. However, there appear to be differences in children’s screen time across countries^19–21^. For example, four to six-year-olds from medium–high SES families in Germany and Spain were reported to have 20–30 min of daily TV time, whereas this estimate was between 30–90 min in Greece, and 60–240 min in Belgium, Poland, and Bulgaria^20^. Thus far, most studies have focused on children from English-speaking countries^22,23^. For example, of the 29 studies concerning screen time in children aged ≤ three years reviewed by Duch and colleagues^22^, 25 were conducted in the US, while only four were conducted in Europe and East Asia. Furthermore, most of the studies reviewed in Duch and colleagues^22^ focused on TV viewing rather than mobile media use. Moreover, available survey estimates of children’s screen time tend to focus on older children: For example, EU Kids Online or the UK’s Office of Communications, which publishes annual survey results of children’s screen time, does not provide data for children under the age of three. Thus, there is a gap in the literature with regards to a more global perspective on toddlers’ screen time, especially given the widespread availability of mobile media devices.

Screen time is strongly embedded in the familial context, with older siblings often showing their younger siblings how to use media devices, helping them choose content, and co-using digital devices or co-viewing^17,24^, but see ^22,25^ for null results]. This sibling influence on screen time entails that toddlers with older siblings are often exposed to content that is targeted at older children^26,27^. Furthermore, there are differences in co-viewing and co-using patterns across early development, with some studies finding that the rate of parent–child co-use of mobile media is highest in children younger than two years and decreases dramatically as the child grows older^13,18^.

Exposure to screen time may be particularly exacerbated in children from lower socio-economic status (SES) families^13,20,23,28–32^. Reports suggest that 97% of children aged six months to four years from low-income backgrounds in the US used mobile devices, and that most children started using them before their first birthday^30^. Caregiver education is also strongly associated with screen time given findings that, among 0–8-year-olds, the average daily screen time varies from 3 h 12 min for children whose caregivers have a high school diploma (or less) to 2 h 24 min for children whose caregivers have some college experience, and 1 h 38 min for children whose caregivers have a college degree^13^. In line with this, a recent large-scale multinational study, on which the present study builds, also reported a negative association between maternal education and young children’s screen time during the first COVID-19 lockdown^33^.

Caregiver behaviours and attitudes also play an important role in young children’s screen time. Caregivers of children under the age of three who reported having longer screen time themselves reported allowing their children more time with smart devices^17^. Caregivers with stronger beliefs about the educational benefits of screen time were more likely to co-use mobile media with their child than to have the child use mobile media alone^34^. One- to four-year-olds were also more likely to have access to their caregiver’s smartphone if caregivers believed that it was important for their child to familiarize themselves with technology^35^.

Taken together, the studies reviewed above suggest that children have access to screens from the first year of life and that screen time is linked to socio-demographic factors such as child age, socioeconomic status of the family, the number of siblings, as well as factors such as caregiver’s screen time and attitudes towards screen time, although these effects appear to vary cross-nationally.

## Children’s screen time during COVID-19 lockdown

Children’s screen time also appears to be modulated by disruptive external influences. For example, several studies reported more screen time among hospitalized children^36,37^. More recently, in addition to pre-existing differences in children’s physical activity and screen time across countries^38^, the COVID-19 pandemic and the associated lockdown measures have triggered an increase in screen time in many regions. In Spain, two out of three children under 48 months used smartphones and tablets daily during COVID-19 lockdown^16^. Table 1 presents the key findings of studies examining lockdown-related increases in screen time among older children across several countries. Taken together, these studies suggest a widespread, immediate, and potentially adverse impact on children’s screen time as a consequence of the COVID-19 lockdown measures in older children between 3 to 18 years of age. But, to our knowledge, no single study has examined the effects of the COVID-19 lockdown and associated measures (i.e., lockdown severity and duration) on younger children’s screen time across countries.

## Lockdown severity across countries

The duration and implementation of lockdowns and social distancing measures introduced to contain the pandemic differed widely across countries, resulting in various degrees of lockdown severity. To account for lockdown severity, the Oxford COVID-19 Government Response Tracker (OxCGRT) project suggested a Stringency Index, based on the following factors: school and childcare closures, workplace closures, cancellation of public events, social contact restrictions, public transport closures, and movement restrictions^39^. The 12 countries participating in this study imposed various degrees of lockdowns for different periods of time (see Table 1, Supplementary Information). Most countries started implementing lockdown measures in March 2020. The two countries that implemented the strictest measures according to the COVID-19 Government Stringency Index were Saudi Arabia and Israel. Although some countries allowed outdoor activities for children whilst maintaining social distancing practices (e.g., Norway and the Netherlands), others did not allow children under the age of 15 to leave the house, even after lockdown measures were eased later in 2020 (e.g., Turkey and Saudi Arabia). Against the background of studies suggesting disruptive environmental influences on children’s screen time^36,37^, differences in lockdown measures are likely associated with children’s screen time, for example, because they limit the activities children can engage in.

## Children’s screen time and language development

Health agency reports suggest that excessive screen time in the early years can be detrimental to early development, e.g.,^40–45^. However, such reports also stress that there is limited work examining the impact of screen time in young children and highlight children’s learning from digital media when caregiver support and scaffolding is provided.

The results of the few studies examining the impact of frequency and duration of screen time on children’s development are mixed. On the one hand, population-based studies suggested a negative association between excessive screen time in early childhood and children’s language development^27^, especially with regards to children’s expressive (but not receptive) vocabulary^46^ (but see^33^ for similar results), as well as a negative association between screen time and children’s receptive vocabulary^47^. On the other hand, a recent meta-analysis found that while increased screen time was associated with lower language skills, quality screen time (educational programs) and caregiver scaffolding during screen time was associated with stronger language skills in children under twelve years of age^48^. Thus, there is a need for further examination of the association between children’s screen time and language development during lockdown.

## The current study

The current study addresses the following three limitations of previous research on young children’s screen time. First, the literature currently lacks a systematic investigation of the factors associated with screen time in children under the age of three. Second, to our knowledge, there is no study of young children’s screen time during lockdown, especially with regards to potential increases in screen time during lockdown relative to pre-lockdown. Third, the literature lacks a global record of young children’s screen time. Thus, more diverse data collected through similar sampling methods are necessary to begin to understand cross-national differences in young children’s screen time. Such comparative research will shed new light on the factors that influence screen time both globally and nationally, and will allow informed recommendations for young children’s screen time. We address this gap by examining screen time during lockdown in a large international sample of 8-to-36-month-olds.

In particular, this study has the following aims: To examine *(i)* the factors associated with young children’s screen time during the first COVID-19 lockdown, *(ii)* whether there was an increase in young children’s screen time during lockdown relative to before the first COVID-19 lockdown and *(iii)* the association between potential increases in screen time during lockdown and vocabulary development.

## Predictions

We predicted that caregiver’s screen time, caregiver’s beliefs about the positive impact of screen time, maternal education level (as a proxy of SES), children’s age, and the severity of the lockdown in the country would be positively associated with children’s screen time. We also predicted an increase in children’s screen time during lockdown, compared to before lockdown. Finally, we predicted that an increase in children’s screen time during lockdown would be negatively associated with vocabulary gains during lockdown.

## Results

### Factors associated with young children’s screen time during lockdown

First, we examined young children’s screen time during lockdown using the COVID-language dataset (^33^, https://osf.io/ty9mn/, (see Model 1 specification and additional model parameters in Supplementary Information (Table 2), n = 1292). The full model was compared to a null model excluding all fixed effects predictors except *SES* as preregistered.

**Table 2:** Factors associated with children’s screen time during lockdown using data from the COVID-language dataset (n = 1292). For the full model output see Table 3 in Supplementary Information.

Term	Estimate	SE	Lower CI	Upper CI	LRT	df	*p*
0|3	− 0.896	0.279	− 1.416	− 0.352			
3|4	− 0.416	0.278	− 0.934	0.126			
4|5	0.530	0.278	0.017	1.084			
5|6	1.854	0.283	1.335	2.456			
6|7	3.343	0.300	2.832	3.970			
7|8	4.634	0.342	4.046	5.408			
8|9	6.219	0.498	5.468	7.514			
Lockdown.severity	− 0.230	0.248	− 0.754	0.247	0.833	1	0.361
Lockdown.duration	0.029	0.084	− 0.137	0.215	0.117	1	0.732
**Caregiver.screentime**	**0.270**	**0.062**	**0.154**	**0.380**	**10.560**	**1**	**0.001**
Siblings	− 0.080	0.059	− 0.207	0.043	1.421	1	0.233
**Age**	**1.008**	**0.096**	**0.839**	**1.219**	**25.449**	**1**	**0.000**
**SES**	− **0.424**	**0.095**	− **0.615**	− **0.237**	**10.689**	**1**	**0.001**

Bold formatting highlights significant predictors in the models presented.

The full-null model comparison was significant, *χ2* = 41.51, *df* = 6, *p* < 0.001, suggesting that at least one (or a combination of one or more) predictors lacking in the null model improved model fit. Table 2 shows the resulting parameter estimates with p-values examining the individual contribution of each of the predictors entered into the model. Note that this model does not include the interaction between *lockdown.severity* and *lockdown.duration* (which was included in the original model) due to this interaction not being significant, *χ2* = 2.83, *df* = 1, *p* = 0.093 (see Table 3, Supplementary Information). We, therefore, report here the results of a reduced model excluding this interaction to examine potential main effects of *lockdown.severity* and *lockdown.duration*.

**Table 3:** Factors associated with children’s screen time during lockdown using data from the COVID-screen dataset (n = 951). For the full model output see Table 5in Supplementary Information.

Term	Estimate	SE	Lower CI	Upper CI	LRT	df	*p*
0|1	− 0.632	0.363	− 1.346	0.034			
1|2	0.162	0.362	− 0.546	0.861			
2|3	1.367	0.365	0.652	2.085			
3|4	2.405	0.371	1.703	3.125			
4|5	3.216	0.380	2.504	3.964			
5|6	4.104	0.401	3.372	4.990			
Lockdown.severity	0.304	0.372	− 0.464	1.059	0.617	1	0.432
Lockdown.duration	0.005	0.135	− 0.346	0.361	0.001	1	0.971
Caregiver.screentime	**0.268**	**0.079**	**0.078**	**0.445**	**6.150**	**1**	**0.013**
Age	**0.608**	**0.104**	**0.419**	**0.806**	**9.229**	**1**	**0.002**
caregiver.affect	**0.665**	**0.119**	**0.437**	**0.905**	**8.477**	**1**	**0.004**

Bold formatting highlights significant predictors in the models presented.

**Figure 1:**
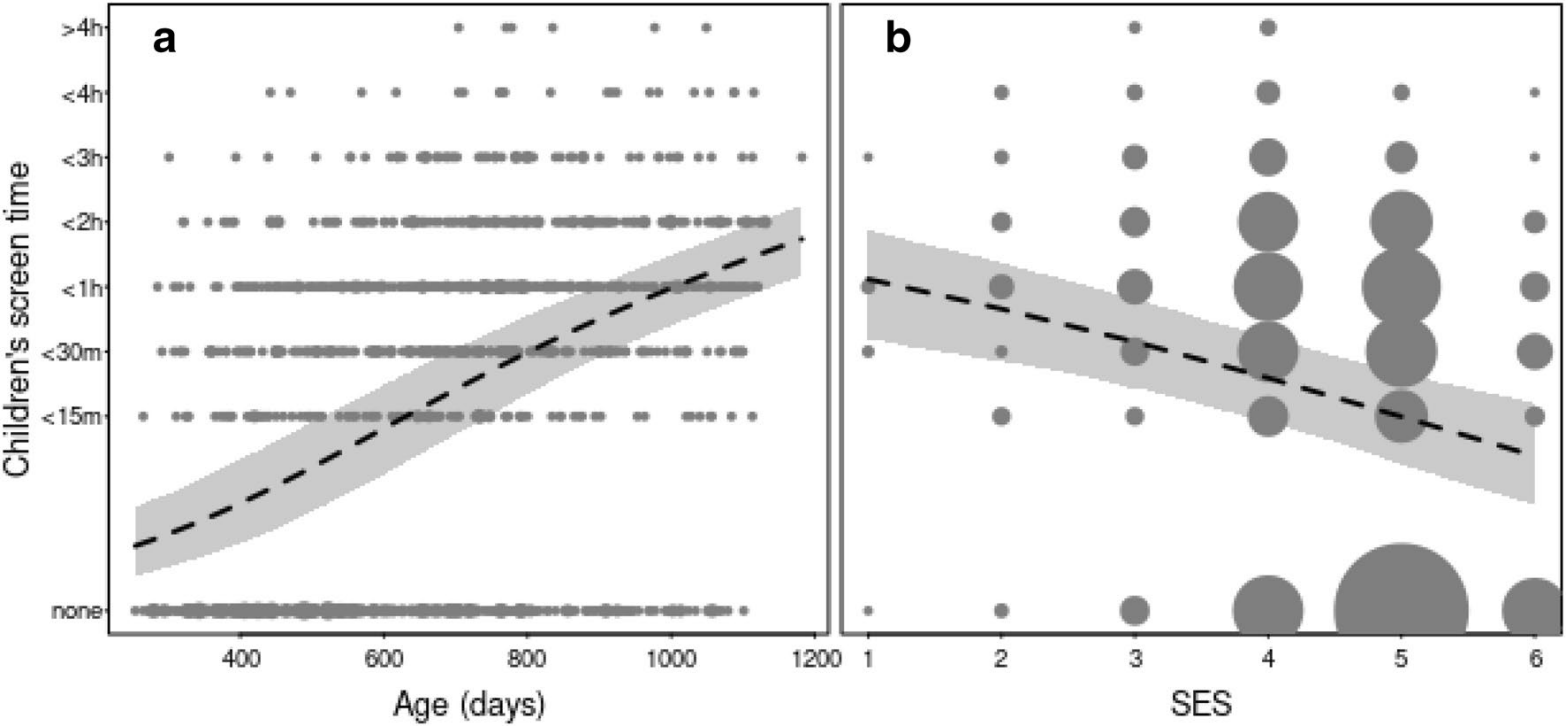
Positive association between children’s screen time and age (in days; panel a) and negative association between children’s screen time and SES (indexed by maternal education; see text for scale; panel b) in the COVID-language dataset (n = 1292). The dashed lines and the shaded area indicate the fitted model and its 95% confidence intervals (with all other terms in the model centered to a mean of zero). The area of the dots is proportionate to the number of the respective observations (range 1–332).

The model summary presented in Table 2 suggests a positive association between *caregiver screen time* and *children’s screen time*, with caregivers who reported having more screen time themselves also reporting their children having more screen time. There was also a positive association between *age* of the child and screen time, with older children having more screen time than younger children (see Fig. 1). Finally, there was a negative association between *SES* and screen time, with caregivers from lower SES families reporting their children having more screen time (see Fig. 1). We found no evidence for associations between screen time and *lockdown.severity*, *lockdown.duration* or the number of *siblings* a child had.

Next, we fitted an additional model (see Model 2 specification and additional model parameters in Table 4 in Supplementary Information, n = 951) including only participants from the COVID-screen dataset who provided information on caregivers’ affective response to children’s screen time (*caregiver.affect*) based on their responses to statements about potential positive or negative side effects of their children’s screen time. We did not include *SES* or *siblings* as a predictor in this model (due to data loss). However, a separate model including *SES* and *siblings* as predictors revealed very similar results to those reported here (see Table 6 in Supplementary Information, Model 2.SES, n = 622). The full model was compared to a null model excluding all predictors as preregistered. The full-null model comparison was significant, *χ2* = 26.31, *df* = 6, *p* < 0.001. Table 3 shows the resulting parameter estimates together with p-values. Note that this model does not include the interaction between *lockdown.severity* and *lockdown.duration* due to this interaction not being significant, *χ2* = 0.079, *df* = 1, *p* = 0.375, see Table 5 in Supplementary Information). We, therefore, report here the results of a reduced model excluding this interaction to examine potential main effects of *lockdown.severity* and *lockdown.duration*.

**Figure 2:**
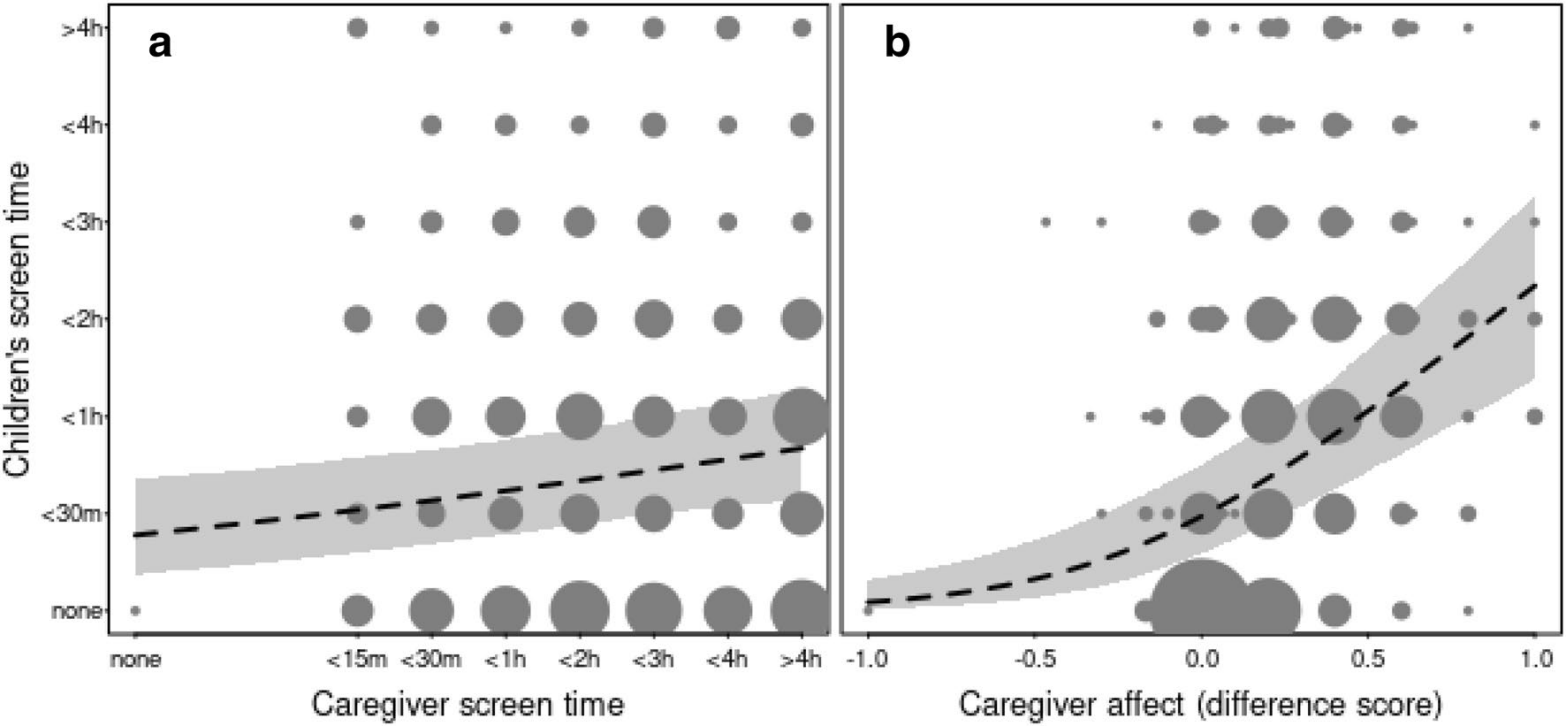
Positive association between children’s screen time and caregiver screen time and between children’s screen time (n= 951; panel a) and caregiver affective response to children’s screen time in the COVID-screen dataset (n = 951; panel b). The dashed lines and the shaded area indicate the fitted model and its 95% confidence intervals (with all other terms in the model centered to a mean of zero). The area of the dots is proportionate to the number of the respective observations (range 1–197).

The model summary presented in Table 3 suggests a positive association between *caregiver screen time* and *children’s screen time* (see Fig. 2), as well as *age* of the child and *screen time*. As in the previous models, we found no obvious associations between *lockdown.severity*, *lockdown.duration* and *siblings* on children’s screen time – while the model including *SES* (see Tables 6 and 7 in Supplementary Information, **Model 2.SES**) replicated the negative association between *SES* and children’s screen time. Importantly, we found a positive association between caregiver’s affective response to screen time and children’s screen time (*caregiver.affect*), with caregivers who reported being more positively inclined towards children’s screen time also reporting their child having more screen time (see Fig. 2).

## Differences in young children’s screen time prior to and during lockdown

We fitted an additional model including only participants from the COVID-screen dataset who provided information on how much screen time their children had access to prior to and the lockdown as well as during the lockdown (see Model 3 specification and additional model parameters in Table 8 in Supplementary Information, n = 953). The full model was compared to a null model excluding all fixed effects predictors except *caregiver.affect* as preregistered.

The full-null model comparison was significant, *χ2* = 32.95, *df* = 8, *p* < 0.001. Tests of individual effects suggested a significant interaction between *lockdown.stage* and *lockdown.duration, χ2* = 4.59, *df* = 1, *p* = 0.032. There were no interactions between *lockdown.stage* and any of the other predictor variables. Model 3 in Supplementary Information (Table 8) shows the resulting parameter estimates together with p-values.

**Figure 3:**
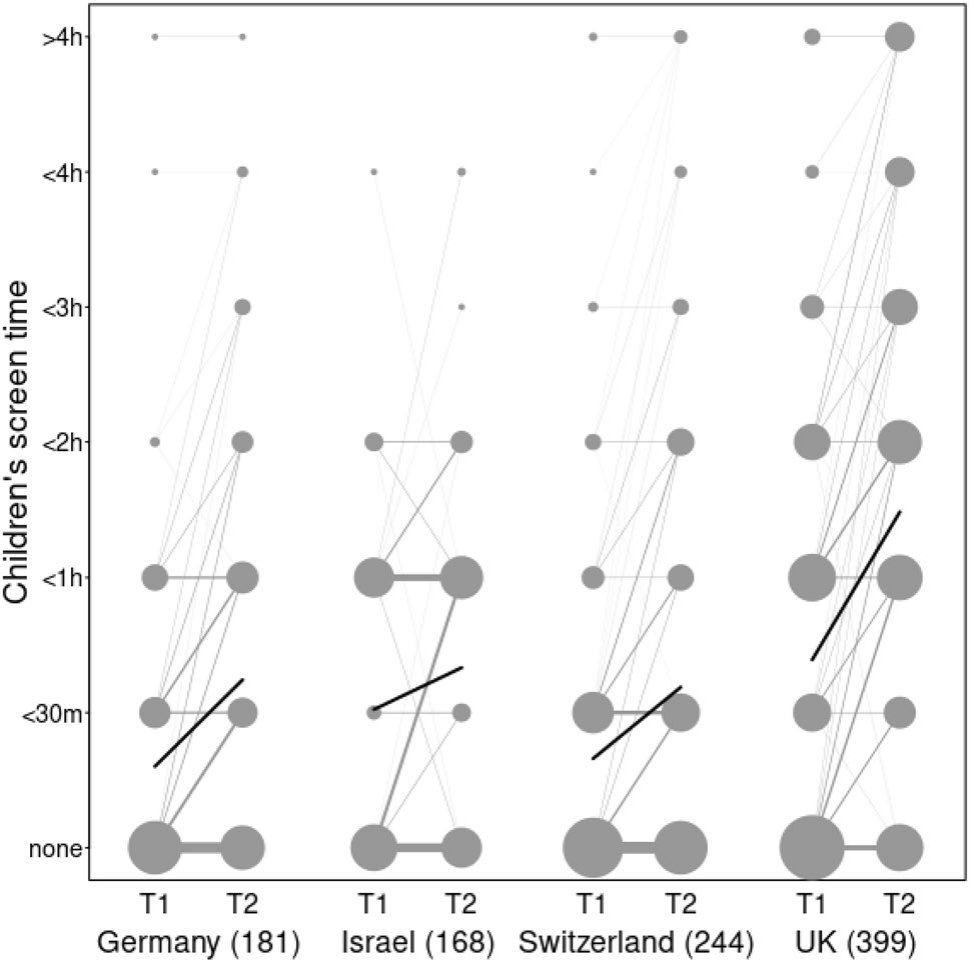
Children's screen time at T1 and T2 of data collection, separated by country, sample size indicated below the x-axis. The area of the dots indicates proportion within a sample, lines connect paired data and line thickness signals proportion such that thicker lines reference more common patterns. The blue overlay line indicates mean change to highlight overall trends in the data.

**Figure 4:**
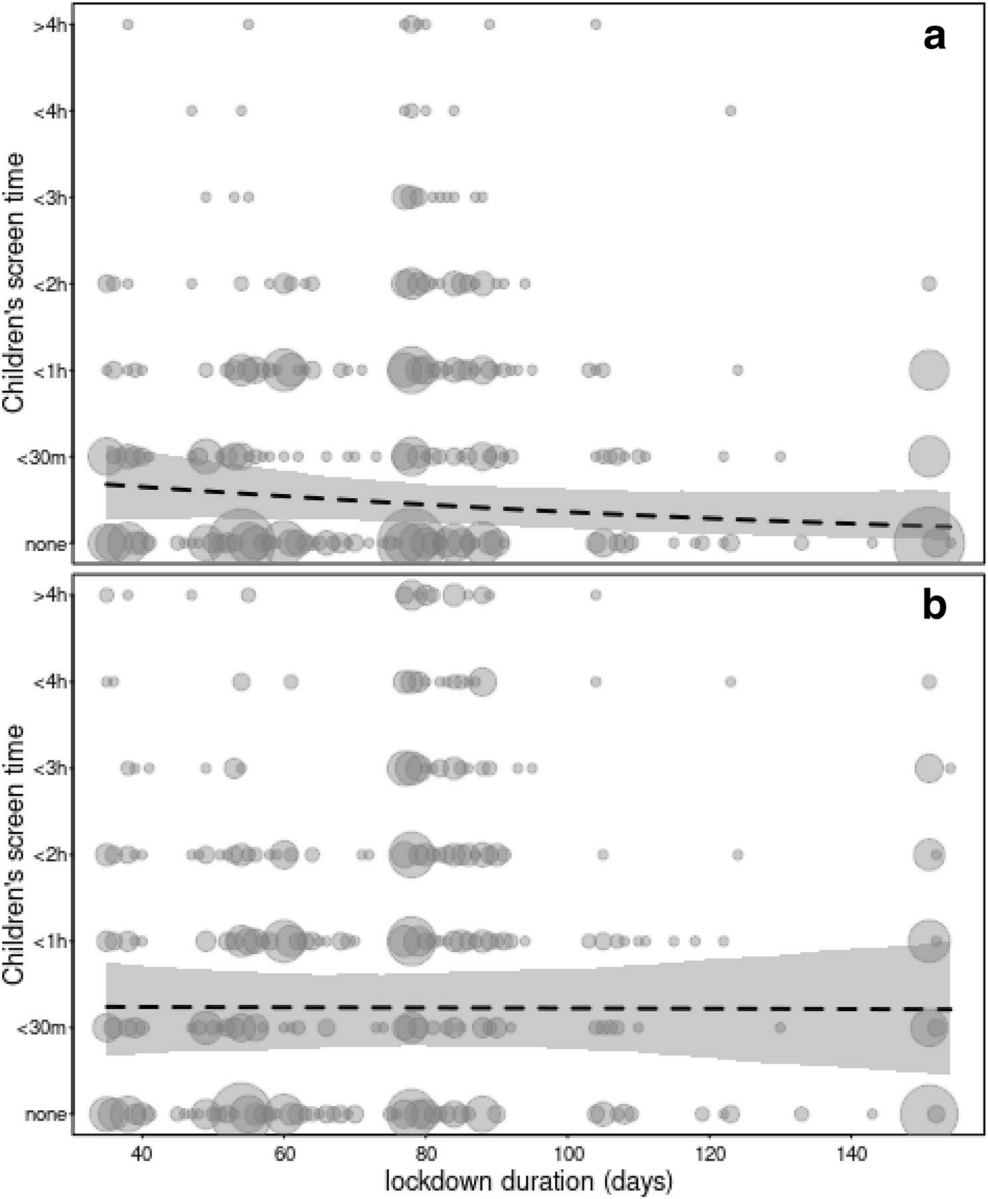
Children's screen time at T1 (panel a) and T2 (panel b) by lockdown duration (in days), the dashed line indicates the model estimates and the shaded area its 95% confidence intervals. The area of the dots is proportionate to the number of the respective observations (range 1–51).

As Fig. 3 suggests, children had more access to screen time during lockdown relative to before the lockdown. Figure 4 depicts the data and the fitted model to illustrate the interaction between *lockdown.stage* (T1, top panel; T2, bottom panel) with *lockdown.duration* and shows that there is a small, but in our models, significant interaction effect (see Table 3) such that longer lockdown durations were associated with a greater increase in screen time. There was no evidence that increases in screen time during lockdown were associated with the other predictors included. We note that as the model is implemented on a reduced dataset, including *SES* and *siblings* as predictors failed to converge.

## Lockdown-related surge in screen time and vocabulary development

Finally, we examined whether lockdown-related increases in children’s screen time impacted children’s vocabulary development, such that those children who were reported to have had more screen time during lockdown were also reported to show smaller gains in vocabulary development during lockdown, separately for children’s receptive and expressive percentile scores (see **Model 4a/b** specification and additional model parameters in Table 9 and 11 in Supplementary Information, n = 117 and 156 respectively). The full models were compared to null models excluding all fixed effects predictors except *SES* as preregistered.

**Figure 5:**
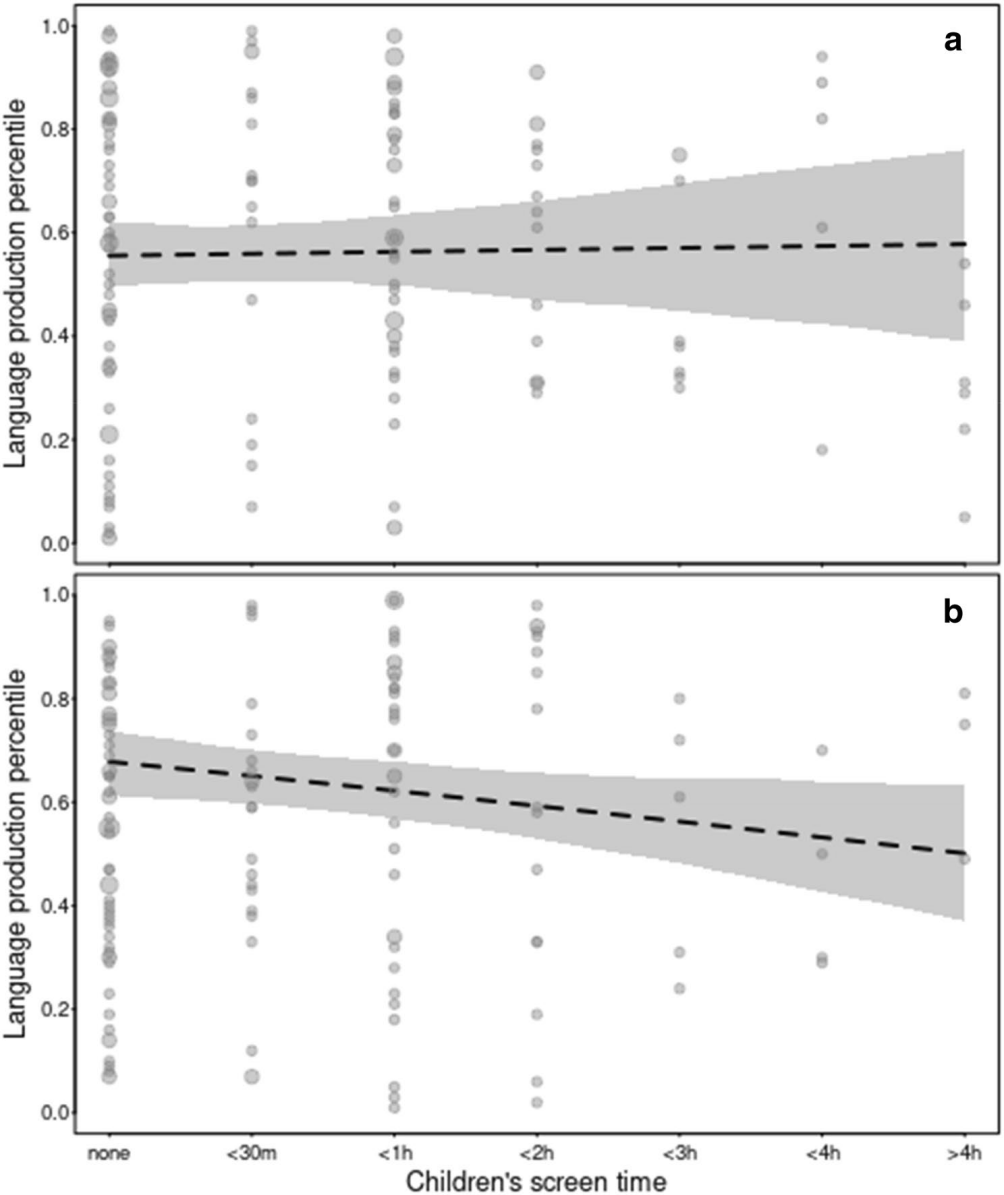
Expressive vocabulary size and children’s screen time before lockdown (T1, top) and during lockdown (T2, bottom). The dashed lines and the shaded area indicate the model estimates and its 95% confidence intervals (with all other terms in the model centered to a mean of zero). The area of the dots is proportionate to the number of the respective observations (range 1–4).

The full-null model comparison for the receptive model was not significant, *χ2* = 13.02, *df* = 7, *p* = 0.072, while the full-null model comparison for the expressive model was significant, *χ2* = 14.92, *df* = 7, *p* = 0.037. We further fitted reduced models including only the interaction between *lockdown.stage* and *screen.time* given that this interaction was near-significant in the full model for expressive vocabulary, *χ2* = 3.52, *df* = 1, *p* = 0.060 (see Table 12 in Supplementary Information). In this reduced model, the interaction between *lockdown.stage* and *screen.time* was significant for expressive vocabulary, *χ2* = 4.63, *df* = 1, *p* = 0.031. While the results with regards to the significant interaction between lockdown.stage*screen.time should be treated with caution due to the marginally non-significant interaction in the full model, they suggest that those children who had smaller increases in screen time during lockdown relative to prior to lockdown were reported to have larger increases in expressive vocabulary during lockdown (see Fig. 5).

## Discussion

This study examined 8–36-month-old children’s screen time during the first COVID-19 lockdown. With regards to the factors associated with young children’s screen time during lockdown, we found that toddlers had exposure to screens from very early on: From as early as eight months of age, *some* children appeared to have regular daily exposure to screens. Nevertheless, we found that screen time increased with age, with older children reported to have more screen time than younger children. This effect was consistent across the two datasets and the literature^11,13,15–18^. Socioeconomic status (SES), as indexed by maternal education, was negatively associated with screen time, with caregivers from lower SES families reporting that their children had more screen time than caregivers from higher SES families, although we note that our sample was biased towards higher SES families. Therefore, this finding may not generalize to the wider population.

We also found a positive association between children’s and caregivers’ screen time consistent with the literature^17^. Probing this relation further, caregivers’ beliefs about the impact of screen time (e.g., that children’s screen time allows them contact with others outside the family, or leads to fights between siblings) was associated with children’s screen time, such that caregivers who were more positively inclined to screen time also reported allowing their children more screen time. This finding may suggest that caregivers’ attitudes towards children’s screen time predict the extent of children’s screen time exposure. However, we note that an alternative explanation of this association may be that those caregivers who were more positively inclined towards children’s screen time were also more likely to report that their child had access to screen time, due to their not viewing this activity negatively. Our results are unable to tease apart these two explanations.

Furthermore, as Fig. 3 suggests, we found that even toddlers were reported to have had more screen time during lockdown relative to the period before lockdown. While this echoes previous results with older children (see Table 1), increases in screen time during lockdown in school-aged children are likely related to enforced online schooling. Here, we found that, despite toddlers having no online schooling requirements, even these youngest members of our societies had increased exposure to screens during lockdown. There was some evidence that this increase in screen time during lockdown was related to the duration of lockdown in specific countries, such that children from countries who enforced longer lockdowns had increased screen time relative to children from countries with shorter lockdowns. This finding highlights how differences in environmental factors, such as, for instance, restrictions during lockdown on activities that families typically engaged in, can impact children’s screen time early in development. Thus, the longer caregivers were at home caring for their children while also working from home - with limited access to other activities to occupy their charges - the more screen time their children reportedly had access to. However, we found no evidence that increases in screen time during lockdown were associated with other sociodemographic characteristics such as SES or the age of the child.

Finally, we discuss the potential impacts of increased screen time on children’s development during lockdown, particularly with regard to children’s vocabulary development. A study on children^33^ at the same age as those reported here found that children’s gains in expressive vocabulary size during lockdown were negatively associated with children’s screen time. Extending these findings, we found tentative evidence for the hypothesis that children who experienced larger increases in screen time during lockdown relative to before lockdown showed smaller increases in their expressive vocabulary during lockdown, such that their language development was on par with, but not exceeding, expected growth. While this finding suggests that abrupt changes in children’s daily lives may have consequences for their language development, we note that these results should be treated with caution due to the exploratory nature of the analyses. Nevertheless, these findings raise questions regarding why screen time, especially with regards to suddenly increased screen time during the lockdown, is negatively associated with language and other developmental milestones. While some explanations for similar findings target the difficulties toddlers face when learning words from screens, others target potential negative effects of screen time with regards to the fact that screen time may displace time spent on other enriching activities. For instance, children who have increased exposure to television, spend less time reading or being read to^49,50^, and have fewer books at home^31^. Our findings could have implications for public health and practice, given the suggestion that increased sedentary screen time during lockdown is negatively associated with children’s language outcomes. However, we advise caution with regards to this suggestion given the tentative nature of the association between language development and increased screen time reported here.

Furthermore, it should be noted that the variables measured in this study, such as screen time and vocabulary size, are subject to self-report biases. During lockdown, many caregivers worked from home and thus had increased opportunities to observe their child, and this increase might have caused the overestimation of screen time and vocabulary size (see^33^ for a similar discussion). In addition, given our use of a 10-point scale to measure screen time, our data cannot speak for the exact number of hours children spent in front of the screen. Finally, we note that we do not report on the content and context of screen time in the current manuscript. This is especially important given that some findings suggest that the quality of screen time may be more strongly associated with developmental outcomes than the quantity of screen time. Indeed, we did collect data on the content (educational, entertainment) of children’s screen time as well as the context of their screen time (alone, with siblings, with caregivers). However, this data was only collected in a handful of countries and there was considerable variability in the exact questions included across countries. Planned country-specific examination of these issues will address these questions further, and may provide further detail into the influence of screen time on early development.

## Conclusions

This study highlights the consequences of the COVID-19 pandemic on early development. On the one hand, we found that toddlers with no online schooling requirements were exposed to more screen time during lockdown relative to prior to lockdown. We also found that this may have been particularly exacerbated in countries with longer lockdowns. On the other hand, we found that factors previously associated with screen time before the lockdown in the literature were associated with screen time during lockdown in the current study (i.e., the age of the child, SES, caregiver screen time and caregiver attitude to screen time). We interpret this in terms of the continuity of the presence of screens in young children’s lives. The COVID-19 pandemic provided us a unique window to explore the changes in children’s lives, and to examine sources of individual as well as cultural differences in screen time in young children. These findings shed light on the way different families view, use, and are affected by screens in both their normal and disrupted lives.

## Methods

This study’s predictions and analyses were pre-registered on the Open Science Framework (https://osf.io/4h7sw/) following data collection and prior to data preprocessing, visualisation and analysis. All materials, anonymized data, and analysis codes are available on the project’s OSF. Deviations from the preregistration and exploratory analyses are highlighted below.

### Participants

Families with children aged between 8 and 36 months were recruited between March and September 2020 by 15 labs across 12 countries (Canada, France, Germany, Israel, Norway, Poland, Russia, Saudi Arabia, Switzerland, Turkey, UK, USA). Participants were recruited through online advertisements on social media, and contacting caregivers registered to babylab databases. In total, data from 2209 children (and their caregivers) were entered into the models reported in this manuscript, 1292 of which were collected in the context of a larger COVID-lockdown study, henceforth referred to as the *COVID-language dataset* (^33^, see data exclusion details below).

In a subset of countries, henceforth referred to as the *COVID-screen dataset* (Germany, Israel, Switzerland, UK), additional data were collected which explicitly aimed at examining children’s screen time during lockdown (*n* = 1323, n after exclusions = 992, see details below; gender information not available for this sample). Not all of the participants who were in the COVID-screen dataset were included in COVID-language dataset (and vice versa) due to these participants not providing data for mandatory questions in that study.

Some of the analyses reported here focus solely on the COVID-screen dataset (n = 1323). Of these, 331 participants were excluded from the analysis for the following reasons: (a) older or younger than the specified age limit in the study (i.e., younger than 8 months or older than 36 months; *n* = 261), (b) participant information not available or conflicting participant information across datasets (*n* = 66) or (c) participants completed the study after the end of data collection (*n* = 4), leaving a total of 992 participants whose data could be entered into the different models. Across all models, we excluded participants who did not provide data for all variables included. The number of participants whose data were entered into each model are highlighted in the results section as well as presented with details of model specification and model output in Supplementary Information B.

Separate analyses examine the participants analysed in the COVID-language dataset (^23^, *n* = 1742, 888 girls), following exclusion of 450 of the original 1742 participants due to conflicts in the data provided (date at which childcare facilities shut down for lockdown later than date of filling in the last questionnaire, *n* = 67), providing responses that deviated from the monotonous scale adopted for the current study (*n* = 353) and/or not providing responses for all variables entered into the model (*n* = 29). Finally, we report analyses including those participants from the COVID-language dataset^33^ for whom we were able to obtain data on both their vocabulary development during lockdown as well as additional data on their screen time during lockdown from the COVID-screen dataset (*n* = 176, 117 of which provided data for the receptive analyses and 156 of which provided data for the expressive vocabulary analyses due to some participants providing data on both children’s receptive and expressive vocabulary size).

### Procedure

As part of a larger global COVID-lockdown study^33^, participants were asked to complete an online questionnaire at the beginning of the first lockdown in March 2020 (T1) and at the end or easing of the lockdown (T2) in their respective countries (between May and September 2020). Some of the participants were only presented with questionnaires at T2 (*n* = 615) and were asked to complete a compiled version of the T1 and T2 questionnaires that included all relevant questions for the current study at this time. T1 and T2 questionnaires also included other variables which are not investigated in this study. The entire study was conducted online. This research was carried out in accordance with the provisions of the World Medical Association Declaration of Helsinki. The project was approved by the ethics committee of the Georg-Elias-Müller Institute for Psychology at the University of Göttingen. Collaborating labs obtained ethical approval from their institutions and each lab followed the ethical guidelines and ethics-review-board protocols of their own institution. All labs obtained informed consent from the legal guardians of the children whose data was included in the study before proceeding with the study. Central data analyses exclusively used depersonalized data.

## Materials

### Time 1 questionnaire

The T1 questionnaire included questions relating to children’s socio-demographic characteristics (i.e., child age, exposure to [different] languages in daily life, number of siblings, caregivers’ native language, caregiver education) and the child’s vocabulary development at T1 (see^33^ for further details). Based on research highlighting the influential role of maternal education on children’s later achievement^51^, we used maternal education as a proxy for socioeconomic status (SES; see Figs. 1 and 2 in the Supplementary Information for distribution of maternal education across the two datasets), measured on a scale from 1 to 6 as follows: 1 (primary school), 2 (high school), 3 (college/University), 4 (Bachelor degree), 5 (Master degree), 6 (Doctoral degree).

#### Vocabulary measure

Children’s receptive and expressive vocabularies were measured using age-appropriate Communicative Development Inventories (CDIs) and their adaptations for the relevant language (or regional variant) of the child. Caregivers were asked to indicate whether their child understands (receptive vocabulary) and/or says (expressive vocabulary) each word in the inventory. Kartushina et al.^33^ transformed the number of words on CDIs to daily percentiles for each language using data from wordbank.stanford.edu^52^ or from norming data collected by authors of that study for this explicit purpose when available. These percentile scores, calculated as described in^33^ constituted our vocabulary measure at T1 in the current study.

### Time 2 questionnaire

The T2 questionnaire included a range of questions examining children’s screen time, vocabulary size at T2 (percentile score similar to T1), caregivers’ screen time and caregivers’ attitude towards children’s screen time, described in more detail below. Some labs included other questions not included here.

#### Children’s screen time

**Figure 6:**
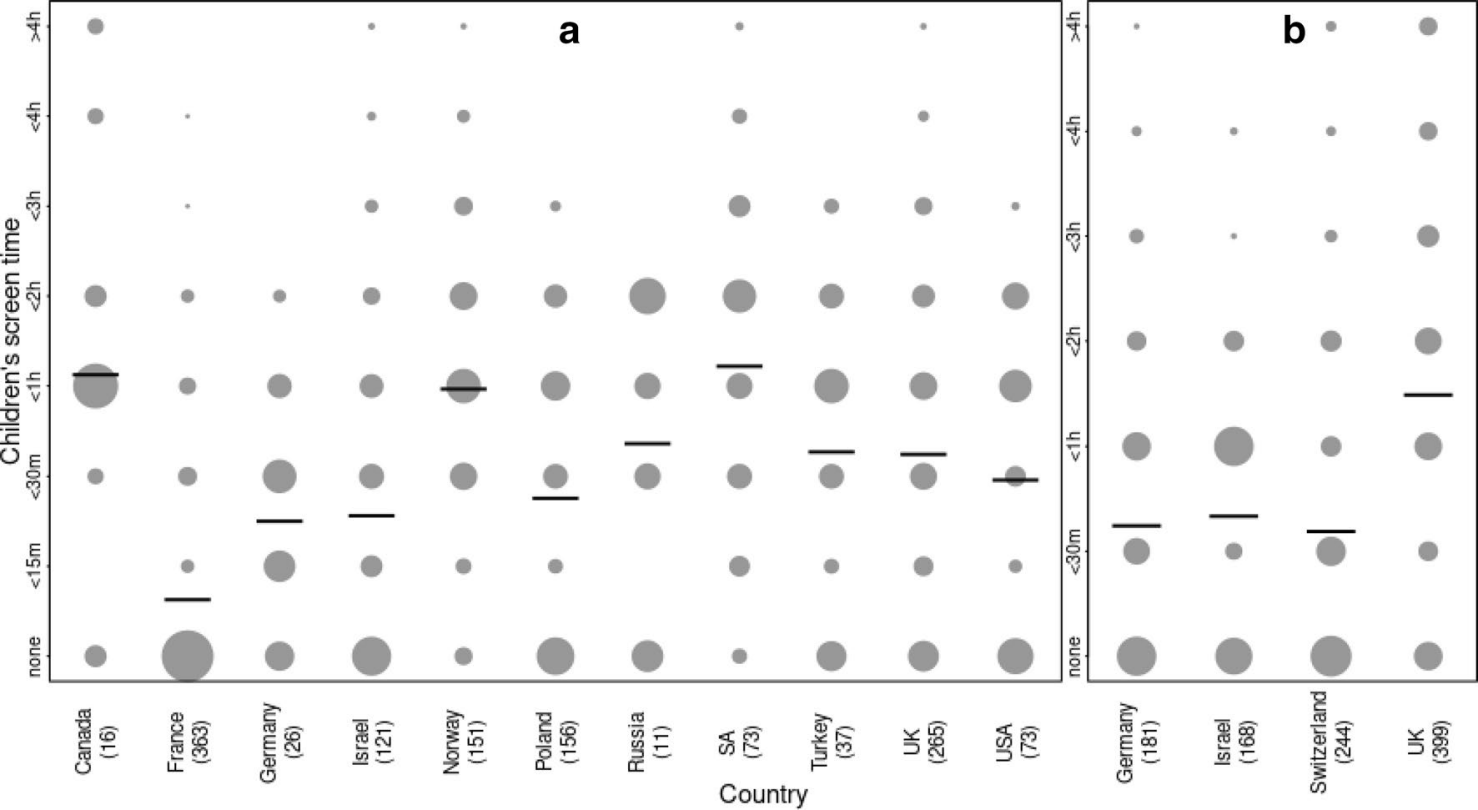
Caregiver reports of children’s screen time (at T2) from the COVID-language dataset (n = 1292, panel a) and the COVID-screen dataset (n = 992; before exclusion for different models, panel b). Overlay lines indicate country-level mean scores.

With some variation between labs, questions targeted the quantity, quality and context of children’s screen time. The current study focused on the quantity of children’s screen time due to considerable data loss with regards to the other descriptives. All labs asked caregivers to rate the amount of time their child spent in watching baby cartoons and shows on any device (e.g., TV, DVD, smartphone) and playing digital baby games. Some questions also separately targeted the quantity of screen time prior to and during lockdown with differences between labs in whether the questions on screen time prior to lockdown were asked at T1 or at T2. Given differences in the specific scales used across different labs, we harmonised the data to a seven-point scale (ranging from “My child never uses these devices” to “More than 4 h per day”) that collapsed across lab-specific scales or time-estimates (see Fig. 6 for distribution of screen time across the participating countries).

#### Caregiver screen time

Caregivers were also asked to report their own screen use on a 10-point scale ranging from “I do not use this type of device” to “More than 6 h per day”. Some labs asked about caregivers’ general use of screens whilst other labs asked about the use of specific devices separately, for example, phones, laptops, and tablets, as well as whether this was in the presence of their child or not, and whether their media use was work related or not. This was harmonised to generate a single value of caregiver screen time on the same seven-point scale as children's screen time (see Figs. 2 and 3 in the Supplementary Information for visualisation of variation in caregiver screen time across the two datasets).

#### Caregiver attitude to children’s screen time

Caregivers were asked to select any perceived positive or negative impacts of their child’s screen use from a list of five positive and six negative possibilities (see Supplementary Information D for options presented to parents and visualisation of caregivers’ perception of children’s screen time). For example, a potential positive impact of media use may be “this allows my child to have contact with family/friends” while a potential negative impact may be “screens lead to sibling fights”.

#### Vocabulary measure

As at T1, we measured children’s receptive and expressive vocabularies using age-appropriate CDIs. Percentile scores based on caregiver responses on the CDIs calculated as described in^33^, constituted our vocabulary measure at T2 in the current study.

### Country-specific lockdown characteristics

#### Lockdown severity

Due to variation in COVID-19 transmission rates and government response, the lockdown restrictions and dates varied between countries. Subsequently, the dates for T1 and T2 data collection also varied across countries. Adapting the Oxford COVID-19 lockdown Stringency Index^39^, we calculated a simple additive lockdown severity index for each country on a three-point scale with one point awarded for each of the following: (1) playground closures, (2) social contact restrictions and (3) restrictions on going outside (see Table 1 in Supplementary Information). We also collected data on whether childcare facilities were shut and whether leisure and eating facilities were closed down but these data were uniform across the countries included here and were excluded from the three-point scale.

#### Lockdown duration

We also calculated the duration of lockdown until the T2 questionnaire was completed in each country or region. Where available, this was based on the number of days between the date on which the T2 questionnaire was filled (the end of lockdown for that family or the end of data collection if lockdown was not yet complete) and the date on which nurseries, preschools and childcare facilities shut down in that region or country. This ranged from 35 to 151 days with a mean of 77 days (see Figs. 5 and 6, Supplementary Information).

### Pre-processing

Following import of the data from the different labs, we identified subtle differences in the scales used by the different labs. We therefore converted the scales provided by the labs to hours and minutes and then reconverted the data to a harmonised scale across labs. This was done by taking the midpoint of the time-range for each value on the scale. Also, as specified in the preregistration, since the response variable was measured on a non-monotonous 0 to 9 scale (varying from number of times per week the child has access to screen time to how many minutes per day the child has access to screen time), we excluded those values (1 and 2) that were on a different scale (number of times per week) relative to the other responses (duration per day). For labs which collected data on screen time across a range of devices or across a variety of digital content (age-appropriate, age-inappropriate), we summed the midpoint time estimates across different devices and/or content to calculate total screen time for each participant. The final harmonised scale across countries entered into the models was the following seven-point scale: 0 (not at all), 1 (0–30 min a day), 2 (30–60 min a day), 3 (1–2 h a day), 4 (2–3 h a day), 5 (3–4 h a day) and 6 (more than 4 h a day). Figure 6 visualizes these data separately for participants from the different countries contributing to the COVID-language dataset and the COVID-screen dataset. With regards to caregiver screen time, we also excluded those values (1 and 2) that were on a different scale (per week) and only retained responses that indicated how much screen time caregivers had per day (see Figs. 3 and 4, Supplementary Information). Finally, with regards to caregivers’ attitude to children’s screen time, caregivers were asked to indicate which of six potentially positive and five potentially negative side-effects of children’s screen time use they perceived: For example, whether screen time led to them having more time for themselves, or to siblings fighting amongst each other. We first examined whether caregivers’ responses to the positive side-effects were correlated with caregivers’ responses to the negative side-effects of screen time. Due to the significant correlation between these two variables, *r* = 0.167, *p* < 0.001, we collapsed the two measures as proposed in the preregistration. In particular, we calculated the percentage of positive side-effects caregivers indicated they agreed with as well as the percentage of negative side-effects caregivers indicated their agreement and then computed the difference between these two as an index of caregiver’s overall view of children’s screen time. Thus, if this measure (henceforth, *caregiver affect*) were positive, it indicates that caregivers indicated they agreed with a greater proportion of the positive side-effects of screen time and vice versa if this measure were negative.

All predictor variables entered into the model were scaled (using the default scale function in R) by calculating the mean and standard deviation of all the values and then scaling each value by subtracting the mean from each value and dividing it by the standard deviation. After this, they had a mean of zero and a standard deviation of one, which eases model convergence.

### Data analysis

First, we examined young children’s screen time during lockdown using the COVID-language dataset^33^ (https://osf.io/ty9mn/). Fixed effects entered into the model were *lockdown.severity* (on a scale of 0 to 3) and *lockdown.duration* (calculated as the number of days since the shutdown of childcare facilities in the city where participants were located and the date the questionnaire was filled in). We also included as fixed effects the number of *siblings*, *age* of the child (in days), and *SES* (as indexed by maternal education) as well as the amount of caregiver screen time. We fitted an ordinal model^53^ using the ordinal package (version 2019–12-10, see references for list of package citations) in R (Version 4.0.3). We included a random intercepts effect for country and all theoretically identifiable random slopes (see Model 1 specification including full random effects structure in Table 2 in Supplementary Information^54^). We initially tried to include parameters for the correlations among random intercepts and slopes, but removed the correlations due to issues with model convergence. All models were fitted on complete datasets. The model reported here was compared to a null model excluding all predictors except *SES* as preregistered. Such a full-null model comparison aims at avoiding “cryptic multiple testing”^55^. The sample analysed with this model comprised a total of 1292 cases from 11 countries.

In addition, using the subset of data which included information on whether caregivers agreed with statements about potential positive or negative side effects of their children’s screen time, we fitted an additional ordinal model (see Model 2 specification including full random effects structure in Table 4 in Supplementary Information) including participants from the COVID-screen dataset who provided information on caregivers’ affective response to children’s screen time (*caregiver.affect*). We did not include SES or number of *siblings* as a predictor in this model (due to data loss). The sample analysed with this model comprised a total of 951 cases from 4 countries. However, a separate model including *SES* and *siblings* as predictors revealed very similar results to those reported here (see Table 6, Supplementary Information). The model reported here was compared to a null model excluding all predictors as preregistered. While we had originally preregistered including whether the child asked for access to screens to the model we did not include this predictor variable due to almost no countries providing data for this variable. The sample analysed with this model comprised a total of 622 cases from 3 countries.

Countries contributing to the COVID-screen dataset (Germany, Israel, Switzerland and the UK) also asked caregivers to provide additional information on how much screen time their children had access to prior to the lockdown as well as during the lockdown. We, therefore, fitted an additional model including all participants who provided information on quantity of screen time prior to and during lockdown (see Model 3 specification including full random effects structure in Table 8 in Supplementary Information). The factor *lockdown.stage* coded for whether the values indicated for the response variable were for the time prior to the lockdown or during lockdown, with the time prior to lockdown as the reference level. The model reported here was compared to a null model excluding all predictors except *caregiver.affect* as preregistered. The sample analysed with this model comprised a total of 953 individuals from 4 countries.

Finally, we examined whether lockdown-related increases in children’s screen time impacted children’s vocabulary development, such that those children who were reported to have had more screen time during lockdown were also reported to show smaller gains in vocabulary development during lockdown. The response variables entered into separate receptive and expressive vocabulary models were children’s receptive and expressive percentile scores respectively (see Model 4a/b specification including full random effects structure in Table 9 and 11 in Supplementary Information). The models reported here were compared to null models excluding all predictors except SES as preregistered. The models on vocabulary development were fitted with a beta error distribution and logit link function^56,57^ and with the function glmmTMB of the equally named package (version 1.1.1;^58^). The samples analysed with these models comprised a total of 234 percentile scores for 132 individuals from two countries (comprehension model) and a total of 312 percentile scores for 172 individuals from three countries (production model). Neither of the two percentile scores was overdispersed (dispersion parameters, comprehension model: 0.588; production model: 0.609).

For all models we determined their stability by dropping levels of the random effects, one at a time, fitting the full model to each of the subset data sets, and finally comparing the range of model estimates obtained for the subsets those obtained for the full data set. This revealed the fixed effects model estimates to be of moderate to good stability (see full model results in Supporting Information C). We determined confidence limits of fixed effects model estimates and fitted values by means of parametric bootstraps (N = 1000). In case of the ordinal models we implemented these with a function written by RM, and for models fitted with the function glmmTMB we used the function simulate of the respective package. We determined the significance of individual fixed effects by dropping them from the respective model, one at a time, and comparing the resulting reduced model with the one from which the fixed effect had been dropped. These comparisons as well as full-null model comparisons utilized a likelihood ratio test^58^. Furthermore, ordinal models make the proportional odds assumption. In essence, this states that effects of the predictors on the probability of the response to exceed a certain value should be the same for all values of the response. We checked whether this assumption was fulfilled and found, in part, indecisive results (see Supplementary Information E for details). Finally, we are aware that, in most of our models, the number of levels associated with the random effects factor *country* was likely too low to reliably estimate its effect. However, with regard to the ordinal models, including *country* as a fixed effect was not possible since lockdown severity did not vary within countries. Hence, we decided to retain the models as preregistered as the best likely approach to estimate the effect of *lockdown.severity* while at the same time controlling for country level variation in toddlers’ screen time. With regard to the models with vocabulary size as the response, we fitted corresponding models into which we included *country* as a fixed rather than a random effect. We did not include any interactions with *country* in these models in order to allow comparability with the original models. The models revealed results very similar to those of the original models (see Supplementary Information F). Furthermore, Supplementary Information G provides details of the random effects by *country* for all of the full models which quantifies the variance attributable to *country*.

## Supplementary Information

Supplementary Information.

## Data Availability

This study’s predictions and analyses were pre-registered on the Open Science Framework (https://osf.io/4h7sw/) following data collection and prior to data preprocessing, visualisation and analysis. All materials, anonymized data, and analysis code are available on the project’s OSF. Deviations from the preregistration and exploratory analyses are highlighted. Identifiable data are securely archived at the Language Archive of the Max Planck Institute in Nijmegen, Netherlands.
