# Supplementary material for: Young children’s screen time during the first COVID-19 lockdown in 12 countries
Source: Sci Rep. 2022 Feb 7;12:2015. doi: 10.1038/s41598-022-05840-5 (PMC8821703; doi:10.1038/s41598-022-05840-5)
Supplement: Supplementary file 1 — Supplementary Information. [file 41598_2022_5840_MOESM1_ESM.docx]

**Supplementary Information A:**

**Table 1.** Lockdown characteristics for participants (and number of participants) from the different countries contributing to the COVID-language dataset. Lockdown duration in brackets indicate the duration of lockdown for participants from the COVID-screen dataset. Participant numbers in brackets refer to participants in the COVID-screen dataset.

| Lab | Country | n | Lockdown severity | Lockdown start date | Mean lockdown duration in days |
| --- | --- | --- | --- | --- | --- |
| ldl | Canada | 17 | 1 | 16.03.2020 | 89 |
| paris_team | France | 467 | 3 | 16.03.2020 | 70 |
| goe | Germany | 186 (181) | 2 | 13.03.2020 | 74 (124) |
| Technion | Israel | 173 (168) | 3 | 15.03.2020 | 50 (60) |
| babyling | Norway | 173 | 2 | 12.03.2020 | 39 |
| multilada | Poland | 223 | 1 | 11.03.2020 | 71 |
| msu | Russia | 17 | 3 | 30.03.2020 | 96 |
| kau-cll | Saudi Arabia | 86 | 3 | 08.03.2020 | 103 |
| hetsl | Switzerland | 244 (244) | 0 | 13.03.2020 | 89 (46) |
| mltlab | Turkey | 40 | 3 | 16.03.2020 | 77 |
| Brookes | UK | 403 (399) | 3 | 23.03.2020 | 87 (81) |
| clcu | UK | 40 | 3 | 23.03.2020 | 86 |
| rhul_baby_lab | UK | 25 | 3 | 23.03.2020 | 70 |
| cogdevlabbyu | USA | 39 | 1 | 17.03.2020 | 45 |
| ilpll | USA | 49 | 1 | 02.04.2020 | 55 |

**Supplementary Information B:** Model specification and parameters

The tables provide the estimates, together with their respective standard error, confidence limits, and significance tests. In tables showing results for the full models, the columns headed 'min' and 'max' depict the range of estimates obtained when excluding levels of the grouping factor (e.g. country) one at a time.

**Table 2. Model 1 (n=1292)**:

screen.time ~ lockdown.severity*lockdown.duration + caregiver.screentime + siblings + age + SES + (1 | country)+(0 + caregiver.screentime | country)+(0 + siblings | country)+(0 + age | country)+(0 + SES | country)+(0 + lockdown.duration | country)

| **term** | **Estimate** | **SE** | **lower CI** | **upper CI** | **LRT** | **df** | **p** | **min** | **max** |
| --- | --- | --- | --- | --- | --- | --- | --- | --- | --- |
| 0\|3 | -0.865 | 0.274 | -1.405 | -0.311 |  |  |  | -1.245 | -0.671 |
| 3\|4 | -0.385 | 0.273 | -0.906 | 0.166 |  |  |  | -0.701 | -0.208 |
| 4\|5 | 0.564 | 0.273 | 0.021 | 1.118 |  |  |  | 0.273 | 0.679 |
| 5\|6 | 1.889 | 0.278 | 1.373 | 2.436 |  |  |  | 1.615 | 2.064 |
| 6\|7 | 3.378 | 0.295 | 2.837 | 4.002 |  |  |  | 3.058 | 3.678 |
| 7\|8 | 4.672 | 0.338 | 4.073 | 5.446 |  |  |  | 4.368 | 4.923 |
| 8\|9 | 6.258 | 0.495 | 5.503 | 7.718 |  |  |  | 5.916 | 6.512 |
| lockdown.severity | -0.245 | 0.242 | -0.697 | 0.237 |  |  |  | -0.337 | -0.040 |
| lockdown.duration | 0.090 | 0.083 | -0.075 | 0.249 |  |  |  | -0.016 | 0.151 |
| **caregiver.screentime** | **0.269** | **0.062** | **0.152** | **0.383** | **10.461** | **1** | **0.001** | **0.209** | **0.306** |
| siblings | -0.080 | 0.059 | -0.201 | 0.027 | 1.395 | 1 | 0.238 | -0.138 | -0.046 |
| **age** | **1.017** | **0.099** | **0.822** | **1.220** | **25.100** | **1** | **0.000** | **0.950** | **1.093** |
| **SES** | **-0.424** | **0.095** | **-0.615** | **-0.236** | **10.711** | **1** | **0.001** | **-0.498** | **-0.390** |
| lockdown.severity:  lockdown.duration | 0.107 | 0.063 | -0.020 | 0.240 | 2.826 | 1 | 0.093 | 0.066 | 0.262 |

All covariates were z-transformed to a mean of zero and a standard deviation (sd) of one. Mean (sd) of the original variables were *lockdown.severity*: 2.484 (0.793); *lockdown.duration*: 70.359 (29.027); *caregiver.screentime*: 5.399 (1.83); *siblings*: 0.259 (0.61); *age*: 671.127 (213.289); *SES*: 4.588 (0.886)

**Table 3. Reduced Model 1 (n=1292):** Excluding non-significant interaction between lockdown.severity and lockdown.duration)

screen.time ~ lockdown.severity + lockdown.duration + caregiver.screentime + siblings + age + SES + (1 | country)+(0 + caregiver.screentime | country)+(0 + siblings | country)+(0 + age | country)+ (0 + SES | country)+(0 + lockdown.duration | country)

| **term** | **Estimate** | **SE** | **lower CI** | **upper CI** | **LRT** | **df** | **p** |
| --- | --- | --- | --- | --- | --- | --- | --- |
| 0\|3 | -0.896 | 0.279 | -1.416 | -0.352 |  |  |  |
| 3\|4 | -0.416 | 0.278 | -0.934 | 0.126 |  |  |  |
| 4\|5 | 0.530 | 0.278 | 0.017 | 1.084 |  |  |  |
| 5\|6 | 1.854 | 0.283 | 1.335 | 2.456 |  |  |  |
| 6\|7 | 3.343 | 0.300 | 2.832 | 3.970 |  |  |  |
| 7\|8 | 4.634 | 0.342 | 4.046 | 5.408 |  |  |  |
| 8\|9 | 6.219 | 0.498 | 5.468 | 7.514 |  |  |  |
| lockdown.severity | -0.230 | 0.248 | -0.754 | 0.247 | 0.833 | 1 | 0.361 |
| lockdown.duration | 0.029 | 0.084 | -0.137 | 0.215 | 0.117 | 1 | 0.732 |
| **caregiver.screentime** | **0.270** | **0.062** | **0.154** | **0.380** | **10.560** | **1** | **0.001** |
| siblings | -0.080 | 0.059 | -0.207 | 0.043 | 1.421 | 1 | 0.233 |
| **age** | **1.008** | **0.096** | **0.839** | **1.219** | **25.449** | **1** | **0.000** |
| **SES** | **-0.424** | **0.095** | **-0.615** | **-0.237** | **10.689** | **1** | **0.001** |

All covariates were z-transformed to a mean of zero and a standard deviation (sd) of one. Mean (sd) of the original variables were *lockdown.severity*: 2.484 (0.793); *lockdown.duration*: 70.359 (29.027); *caregiver.screentime*: 5.399 (1.83); *siblings*: 0.259 (0.61); *age*: 671.127 (213.289); *SES*: 4.588 (0.886)

**Table 4. Model 2 (n=951)**:

screen.time ~ lockdown.severity*lockdown.duration + caregiver.screentime + age + caregiver.affect + (1 | country)+(0 + caregiver.screentime | country)+(0 + age | country)+(0 + age | country)+(0 + caregiver.affect | country)

| **term** | **Estimate** | **SE** | **lower CI** | **upper CI** | **LRT** | **df** | **p** | **min** | **max** |
| --- | --- | --- | --- | --- | --- | --- | --- | --- | --- |
| 0\|1 | -0.561 | 0.342 | -1.259 | 0.097 |  |  |  | -0.905 | -0.133 |
| 1\|2 | 0.234 | 0.341 | -0.457 | 0.896 |  |  |  | -0.040 | 0.689 |
| 2\|3 | 1.439 | 0.344 | 0.768 | 2.127 |  |  |  | 0.900 | 2.116 |
| 3\|4 | 2.476 | 0.351 | 1.783 | 3.210 |  |  |  | 1.881 | 3.345 |
| 4\|5 | 3.286 | 0.361 | 2.554 | 4.035 |  |  |  | 2.716 | 4.133 |
| 5\|6 | 4.175 | 0.382 | 3.431 | 4.993 |  |  |  | 3.584 | 5.121 |
| lockdown.severity | 0.482 | 0.396 | -0.360 | 1.355 |  |  |  | 0.084 | 1.116 |
| lockdown.duration | 0.036 | 0.138 | -0.372 | 0.398 |  |  |  | -0.240 | 0.105 |
| **caregiver.screentime** | **0.266** | **0.079** | **0.087** | **0.445** | **6.122** | **1** | **0.013** | 0.240 | 0.287 |
| **age** | **0.604** | **0.104** | **0.410** | **0.812** | **9.189** | **1** | **0.002** | 0.511 | 0.723 |
| **caregiver.affect** | **0.664** | **0.118** | **0.437** | **0.909** | **8.529** | **1** | **0.003** | 0.534 | 0.784 |
| lockdown.severity:  lockdown.duration | 0.246 | 0.277 | -0.450 | 0.918 | 0.788 | 1 | 0.375 | -0.227 | 0.493 |

All covariates were z-transformed to a mean of zero and a standard deviation (sd) of one. Mean (sd) of the original variables were *lockdown.severity*: 2.053 (1.259); *lockdown.duration*: 77.278 (30.796); *caregiver.screentime*: 6.55 (1.854); *age*: 22.22 (6.428); *caregiver.affect*: 0.215 (0.233)

**Table 5. Reduced Model 2 (n=951):** Excluding non-significant interaction between lockdown.severity and lockdown.duration)

screen.time ~ lockdown.severity+lockdown.duration + caregiver.screentime + age + caregiver.affect + (1 | country)+(0 + caregiver.screentime | country)+(0 + age | country)+(0 + age | country)+(0 + caregiver.affect | country)

| **term** | **Estimate** | **SE** | **lower CI** | **upper CI** | **LRT** | **df** | **p** |
| --- | --- | --- | --- | --- | --- | --- | --- |
| 0\|1 | -0.632 | 0.363 | -1.346 | 0.034 |  |  |  |
| 1\|2 | 0.162 | 0.362 | -0.546 | 0.861 |  |  |  |
| 2\|3 | 1.367 | 0.365 | 0.652 | 2.085 |  |  |  |
| 3\|4 | 2.405 | 0.371 | 1.703 | 3.125 |  |  |  |
| 4\|5 | 3.216 | 0.380 | 2.504 | 3.964 |  |  |  |
| 5\|6 | 4.104 | 0.401 | 3.372 | 4.990 |  |  |  |
| lockdown.severity | 0.304 | 0.372 | -0.464 | 1.059 | 0.617 | 1 | 0.432 |
| lockdown.duration | 0.005 | 0.135 | -0.346 | 0.361 | 0.001 | 1 | 0.971 |
| **caregiver.screentime** | **0.268** | **0.079** | **0.078** | **0.445** | **6.150** | **1** | **0.013** |
| **age** | **0.608** | **0.104** | **0.419** | **0.806** | **9.229** | **1** | **0.002** |
| **caregiver.affect** | **0.665** | **0.119** | **0.437** | **0.905** | **8.477** | **1** | **0.004** |

All covariates were z-transformed to a mean of zero and a standard deviation (sd) of one. Mean (sd) of the original variables were *lockdown.severity*: 2.053 (1.259); *lockdown.duration*: 77.278 (30.796); *caregiver.screentime*: 6.55 (1.854); *age*: 22.22 (6.428); *caregiver.affect*: 0.215 (0.233)

**Table 6. Model 2.SES (n=622):**

screen.time ~ lockdown.severity*lockdown.duration + caregiver.screentime + age + caregiver.affect + SES + siblings + (1 | Country)+(0 + caregiver.screentime | Country)+(0 + age | Country)+ (0 + caregiver.affect | Country) )+(0 + SES | country) )+(0 + siblings | country)

| **term** | **Estimate** | **SE** | **lower CI** | **upper CI** | **LRT** | **df** | **p** | **min** | **max** |
| --- | --- | --- | --- | --- | --- | --- | --- | --- | --- |
| 0\|1 | -0.799 | 0.451 | -1.728 | 0.067 |  |  |  | -1.535 | -0.396 |
| 1\|2 | -0.075 | 0.450 | -0.972 | 0.781 |  |  |  | -0.732 | 0.554 |
| 2\|3 | 1.184 | 0.452 | 0.264 | 2.103 |  |  |  | 0.227 | 2.381 |
| 3\|4 | 2.211 | 0.459 | 1.263 | 3.122 |  |  |  | 1.214 | 3.752 |
| 4\|5 | 3.024 | 0.469 | 2.064 | 3.998 |  |  |  | 2.054 | 4.406 |
| 5\|6 | 3.937 | 0.489 | 2.959 | 5.108 |  |  |  | 2.965 | 5.698 |
| lockdown.severity | 0.502 | 0.432 | -0.282 | 1.483 |  |  |  | -0.137 | 0.854 |
| lockdown.duration | 0.112 | 0.249 | -0.571 | 0.748 |  |  |  | -0.071 | 0.970 |
| **caregiver.screentime** | **0.304** | **0.099** | **0.086** | **0.549** | **4.674** | **1** | **0.031** | **0.162** | **0.316** |
| siblings | 0.157 | 0.083 | -0.017 | 0.345 | 2.089 | 1 | 0.148 | -0.004 | 0.159 |
| **age** | **0.551** | **0.147** | **0.251** | **0.834** | **5.126** | **1** | **0.024** | **0.417** | **0.582** |
| **caregiver.affect** | **0.645** | **0.081** | **0.492** | **0.832** | **9.842** | **1** | **0.002** | **0.594** | **0.918** |
| **SES** | **-0.294** | **0.083** | **-0.479** | **-0.121** | **4.311** | **1** | **0.038** | **-0.357** | **-0.173** |
| lockdown.severity:  lockdown.duration | 0.072 | 0.187 | -0.359 | 0.524 | 0.148 | 1 | 0.701 | -0.026 | 0.008 |

All covariates were z-transformed to a mean of zero and a standard deviation (sd) of one. Mean (sd) of the original variables were lockdown.severity: 2.412 (1.192); *lockdown.duration*: 70.357 (15.666); *caregiver.screentime*: 6.42 (1.903); *siblings*: 0.646 (0.72); *age*: 21.677 (6.533); *caregiver.affect*: 0.242 (0.244); SES: 4.365 (1.039). Model stability was determined through a series of non-mixed models since the data set comprised only three countries.

**Table 7. Reduced Model2.SES (n=622):** Excluding non-significant interaction between lockdown.severity and lockdown.duration)

screen.time ~ lockdown.severity + lockdown.duration + caregiver.screentime + age + caregiver.affect + SES + siblings + (1 | Country)+(0 + caregiver.screentime | Country)+(0 + age | Country)+ (0 + caregiver.affect | Country) )+(0 + SES | country) )+(0 + siblings | country)

| **term** | **Estimate** | **SE** | **lower CI** | **upper CI** | **LRT** | **df** | **p** |
| --- | --- | --- | --- | --- | --- | --- | --- |
| 0\|1 | -0.832 | 0.468 | -1.830 | 0.086 |  |  |  |
| 1\|2 | -0.108 | 0.466 | -1.074 | 0.790 |  |  |  |
| 2\|3 | 1.150 | 0.469 | 0.197 | 2.058 |  |  |  |
| 3\|4 | 2.178 | 0.475 | 1.262 | 3.141 |  |  |  |
| 4\|5 | 2.991 | 0.484 | 2.051 | 3.992 |  |  |  |
| 5\|6 | 3.904 | 0.504 | 2.913 | 4.998 |  |  |  |
| lockdown.severity | 0.431 | 0.409 | -0.361 | 1.339 | 0.981 | 1 | 0.322 |
| lockdown.duration | 0.077 | 0.230 | -0.500 | 0.680 | 0.114 | 1 | 0.736 |
| **caregiver.screentime** | **0.305** | **0.099** | **0.098** | **0.543** | **4.713** | **1** | **0.030** |
| siblings | 0.155 | 0.083 | -0.011 | 0.333 | 1.977 | 1 | 0.160 |
| **age** | **0.549** | **0.144** | **0.278** | **0.834** | **5.206** | **1** | **0.023** |
| **caregiver.affect** | **0.645** | **0.081** | **0.493** | **0.819** | **9.717** | **1** | **0.002** |
| **SES** | **-0.291** | **0.083** | **-0.456** | **-0.111** | **4.200** | **1** | **0.040** |

All covariates were z-transformed to a mean of zero and a standard deviation (sd) of one. Mean (sd) of the original variables were *lockdown.severity*: 2.412 (1.192); *lockdown.duration*: 70.357 (15.666); *caregiver.screentime*: 6.42 (1.903); *siblings*: 0.646 (0.72); *age*: 21.677 (6.533); *caregiver.affect*: 0.242 (0.244); *SES*: 4.365 (1.039)

**Table 8. Model 3 (n=953)**:

screen.time ~ lockdown.stage*(caregiver.affect + age + lockdown.severity+ lockdown.duration) + (1| Country)+(0+lockdown.stage| Country)+(0+age| Country)+(0+caregiver.affect| Country)+(0+I(lockdown.stage*age)| Country)+(0+I(lockdown.stage*caregiver.affect)| Country)+(1| subject)

| **term** | **Estimate** | **SE** | **lower CI** | **upper CI** | **LRT** | **df** | **p** | **min** | **max** |
| --- | --- | --- | --- | --- | --- | --- | --- | --- | --- |
| 0\|1 | 0.549 | 0.365 | -0.139 | 1.368 |  |  |  | -0.015 | 1.570 |
| 1\|2 | 2.406 | 0.377 | 1.707 | 3.269 |  |  |  | 2.167 | 3.733 |
| 2\|3 | 5.074 | 0.411 | 4.275 | 5.986 |  |  |  | 4.406 | 7.053 |
| 3\|4 | 7.096 | 0.448 | 6.223 | 8.158 |  |  |  | 6.438 | 9.299 |
| 4\|5 | 8.565 | 0.483 | 7.609 | 9.756 |  |  |  | 8.045 | 10.558 |
| 5\|6 | 10.044 | 0.535 | 8.986 | 11.309 |  |  |  | 9.546 | 12.262 |
| lockdown.stage | 1.930 | 0.324 | 1.335 | 2.519 |  |  |  | 1.340 | 2.676 |
| lockdown.severity | 0.733 | 0.378 | 0.006 | 1.488 |  |  |  | 0.232 | 2.371 |
| lockdown.duration | -0.456 | 0.255 | -1.093 | 0.144 |  |  |  | -1.289 | -0.107 |
| age | 1.155 | 0.213 | 0.769 | 1.619 |  |  |  | 0.891 | 1.682 |
| caregiver.affect | 1.334 | 0.250 | 0.872 | 1.877 |  |  |  | 0.980 | 1.915 |
| lockdown.stage:  lockdown.severity | -0.139 | 0.331 | -0.798 | 0.558 | 0.179 | 1 | 0.672 | -0.444 | 0.695 |
| **lockdown.stage:**  **lockdown.duration** | **0.439** | **0.206** | **-0.050** | **0.982** | **4.589** | **1** | **0.032** | **0.036** | **1.593** |
| lockdown.stage:age | 0.218 | 0.113 | 0.001 | 0.468 | 2.388 | 1 | 0.122 | 0.180 | 0.294 |
| lockdown.stage:caregiver.affect | 0.233 | 0.146 | -0.037 | 0.533 | 1.964 | 1 | 0.161 | 0.153 | 0.422 |

All covariates were z-transformed to a mean of zero and a standard deviation (sd) of one. Mean (sd) of the original variables were *lockdown.duration*: 77.387 (30.864); *lockdown.severity*: 2.052 (1.257); *age*: 22.211 (6.426); *caregiver.affect*: 0.214 (0.233). *lockdown.stage* was dummy coded with pre-lockdown being the reference category. p-values not shown are of very limited interpretability.

**Table 9. Model 4a (n=117)**:

receptive vocabulary size ~ lockdown.stage*(screen.time + age + caregiver.affect) + SES + (1| Country)+(0+lockdown.stage | Country)+(0+screen.time| Country)+(0+SES| Country)+ (0+age| Country)+(0+caregiver.affect| Country)+ (0+I(lockdown.stage*screen.time)| Country)+ (0+I(lockdown.stage*age)| Country)+(0+I(lockdown.stage*caregiver.affect)| Country)+ (1| subject)

| **term** | **Estimate** | **SE** | **lower CI** | **upper CI** | **LRT** | **df** | **p** | **min** | **max** |
| --- | --- | --- | --- | --- | --- | --- | --- | --- | --- |
| (Intercept) | 0.278 | 0.375 | -0.468 | 1.013 |  |  |  | -0.326 | 0.717 |
| lockdown.stage | 0.332 | 0.081 | 0.163 | 0.501 |  |  |  | 0.189 | 0.370 |
| screen.time | 0.094 | 0.105 | -0.113 | 0.328 |  |  |  | 0.047 | 0.137 |
| age | -0.100 | 0.133 | -0.356 | 0.163 |  |  |  | -0.287 | -0.039 |
| caregiver.affect | -0.127 | 0.134 | -0.378 | 0.170 |  |  |  | -0.178 | -0.095 |
| SES | 0.098 | 0.127 | -0.188 | 0.391 | 0.588 | 1 | 0.443 | 0.075 | 0.296 |
| lockdown.stage:  screen.time | -0.165 | 0.079 | -0.339 | 0.001 | 3.837 | 1 | 0.050 | -0.196 | -0.131 |
| lockdown.stage:age | 0.087 | 0.084 | -0.082 | 0.259 | 1.068 | 1 | 0.301 | 0.058 | 0.176 |
| lockdown.stage:  caregiver.affect | 0.064 | 0.067 | -0.077 | 0.211 | 0.932 | 1 | 0.334 | -0.122 | 0.099 |

All covariates were z-transformed to a mean of zero and a standard deviation (sd) of one. Mean (sd) of the original variables were *screen.time*: 1.59 (1.686); *age*: 16.44 (1.675); *caregiver.affect*: 0.238 (0.219); *SES*: 4.368 (0.875). *lockdown.stage* was dummy coded with pre-lockdown being the reference category. p-values not shown are of very limited interpretability.

**Table 10. Reduced Model 4a (n=117)**: Excluding non-significant interaction between lockdown.stage and age and caregiver.affect)

receptive vocabulary size ~ lockdown.stage*screen.time + age + caregiver.affect + SES + (1| Country)+(0+lockdown.stage | Country)+(0+screen.time| Country)+(0+SES| Country)+ (0+age| Country)+(0+caregiver.affect| Country)+ (0+(lockdown.stage*screen.time)| Country)+(1| subject)

| **term** | **Estimate** | **SE** | **lower CI** | **upper CI** | **LRT** | **df** | **p** |
| --- | --- | --- | --- | --- | --- | --- | --- |
| (Intercept) | 0.267 | 0.364 | -0.499 | 1.014 |  |  |  |
| lockdown.stage | 0.343 | 0.080 | 0.190 | 0.499 |  |  |  |
| screen.time | 0.111 | 0.105 | -0.124 | 0.325 |  |  |  |
| SES | 0.108 | 0.127 | -0.181 | 0.356 | 0.714 | 1 | 0.398 |
| caregiver.affect | -0.091 | 0.129 | -0.360 | 0.209 | 0.498 | 1 | 0.480 |
| age | -0.032 | 0.116 | -0.269 | 0.209 | 0.076 | 1 | 0.783 |
| lockdown.stage:screen.time | -0.145 | 0.078 | -0.308 | 0.011 | 3.160 | 1 | 0.075 |

All covariates were z-transformed to a mean of zero and a standard deviation (sd) of one. Mean (sd) of the original variables were *screen.time*: 1.59 (1.686); *age*: 16.44 (1.675); *caregiver.affect*: 0.238 (0.219); *SES*: 4.368 (0.875). *lockdown.stage* was dummy coded with pre-lockdown being the reference category. p-values not shown are of very limited interpretability

**Table 11. Model 4b (n=156)**:

expressive vocabulary size ~ lockdown.stage*(screen.time + age + caregiver.affect) + SES + (1| Country)+(0+lockdown.stage | Country)+(0+screen.time| Country)+(0+SES| Country)+ (0+age| Country)+(0+caregiver.affect| Country)+ (0+I(lockdown.stage*screen.time)| Country)+ (0+I(lockdown.stage*age)| Country)+(0+I(lockdown.stage*caregiver.affect)| Country)+ (1| subject)

| **term** | **Estimate** | **SE** | **lower CI** | **upper CI** | **LRT** | **df** | **p** | **min** | **max** |
| --- | --- | --- | --- | --- | --- | --- | --- | --- | --- |
| (Intercept) | 0.240 | 0.116 | 0.004 | 0.482 |  |  |  | 0.024 | 0.297 |
| lockdown.stagenow | 0.321 | 0.091 | 0.143 | 0.508 |  |  |  | 0.200 | 0.384 |
| screen.time | 0.023 | 0.111 | -0.189 | 0.231 |  |  |  | -0.277 | 0.087 |
| caregiver.affect | 0.061 | 0.113 | -0.179 | 0.272 |  |  |  | 0.021 | 0.279 |
| age | 0.213 | 0.115 | -0.003 | 0.434 |  |  |  | -0.095 | 0.353 |
| SES | -0.032 | 0.103 | -0.236 | 0.179 | 0.095 | 1 | 0.758 | -0.082 | 0.002 |
| lockdown.stagenow:  screen.time | -0.191 | 0.100 | -0.405 | 0.008 | 3.524 | 1 | 0.060 | -0.270 | -0.123 |
| lockdown.stagenow:  caregiver.affect | -0.096 | 0.083 | -0.265 | 0.083 | 1.319 | 1 | 0.251 | -0.246 | -0.045 |
| lockdown.stagenow:  age | -0.036 | 0.085 | -0.228 | 0.124 | 0.179 | 1 | 0.672 | -0.060 | 0.057 |

All covariates were z-transformed to a mean of zero and a standard deviation (sd) of one. Mean (sd) of the original variables were *screen.time*: 1.59 (1.686); *age*: 16.44 (1.675); *caregiver.affect*: 0.238 (0.219); SES: 4.368 (0.875). *lockdown.stage* was dummy coded with pre-lockdown being the reference category. p-values not shown are of very limited interpretability.

**Table 12. Reduced model (n=156):** Excluding non-significant interaction between lockdown.stage and age and caregiver.affect):

expressive vocabulary size ~ lockdown.stage*screen.time + age + caregiver.affect + SES + (1| Country)+(0+lockdown.stage | Country)+(0+screen.time| Country)+(0+SES| Country)+ (0+age| Country)+(0+caregiver.affect| Country)+ (0+(lockdown.stage*screen.time)| Country)+(1| subject)

| **term** | **Estimate** | **SE** | **lower CI** | **upper CI** | **LRT** | **df** | **p** |
| --- | --- | --- | --- | --- | --- | --- | --- |
| (Intercept) | 0.245 | 0.116 | 0.021 | 0.489 |  |  |  |
| lockdown.stage | 0.323 | 0.091 | 0.123 | 0.506 |  |  |  |
| screen.time | 0.024 | 0.111 | -0.202 | 0.263 |  |  |  |
| SES | -0.037 | 0.103 | -0.228 | 0.159 | 0.131 | 1 | 0.718 |
| caregiver.affect | 0.007 | 0.102 | -0.204 | 0.204 | 0.004 | 1 | 0.947 |
| age | 0.188 | 0.100 | -0.017 | 0.393 | 1.870 | NA | 0.062 |
| **lockdown.stage:**  **screen.time** | **-0.217** | **0.096** | **-0.418** | **-0.007** | **4.633** | **1** | **0.031** |

All covariates were z-transformed to a mean of zero and a standard deviation (sd) of one. Mean (sd) of the original variables were *screen.time*: 1.59 (1.686); *age*: 16.44 (1.675); *caregiver.affect*: 0.238 (0.219); SES: 4.368 (0.875). *lockdown.stage* was dummy coded with pre being the reference category. The significance test of *age* is based on Wald’s z-approximation since the respective reduced model did not converge. p-values not shown are of very limited interpretability.

**Supplementary Information C:** Additional Figures

***Figure 1.*** Maternal education as a proxy for socioeconomic status of the children contributing to the COVID-language dataset. This was measured on a scale from 1 to 6 as follows: 1 (primary school), 2 (high school), 3 (college/University), 4 (Bachelor degree), 5 (Master degree), 6 (Doctoral degree). Circle size refers to proportions within a country, solid black lines highlight trends in the data.


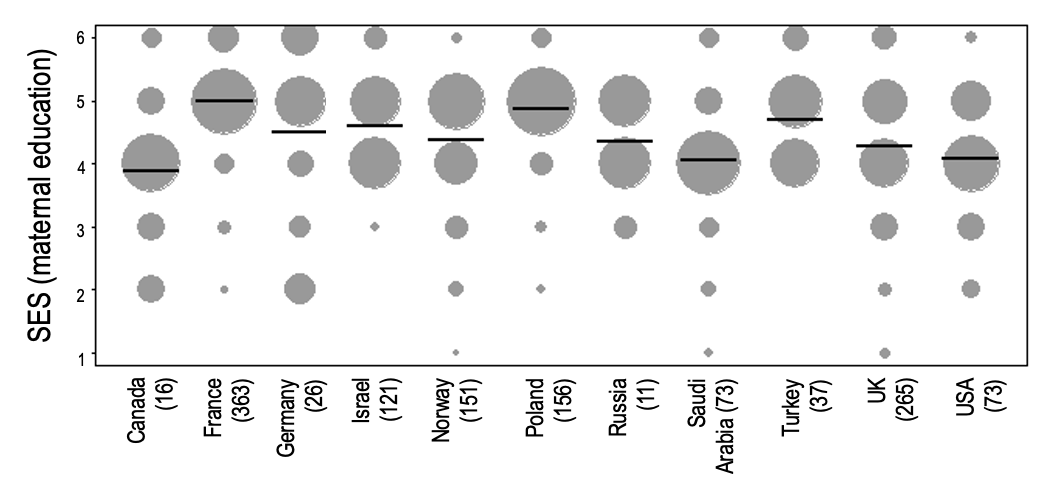


***Figure 2.*** Maternal education as a proxy for socioeconomic status of the children contributing to the COVID-screen dataset. This was measured on a scale from 1 to 6 as follows: 1 (primary school), 2 (high school), 3 (college/University), 4 (Bachelor degree), 5 (Master degree), 6 (Doctoral degree). Circle size refers to proportions within a country, solid black lines highlight trends in the data.

**
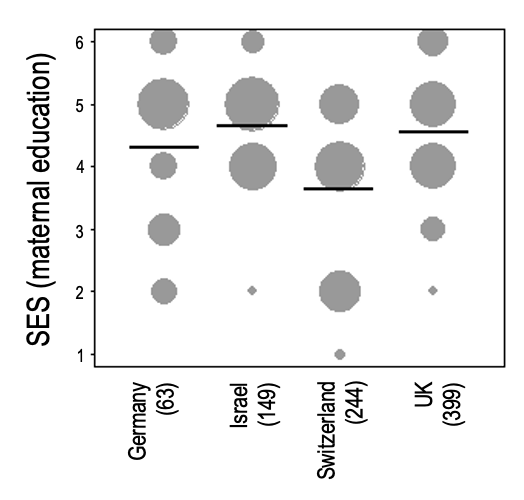
**

***Figure 3.*** Caregivers’ reports of their own screen time using data from the COVID-language dataset. Note that data points that did not fall in the monotonous scale pre-registered for the current study (i.e., duration of screen time per week (1-2/w and 3-4/w) were excluded from the analysis. Circle size refers to proportions within a country, solid black lines indicate means to highlight trends in the data.


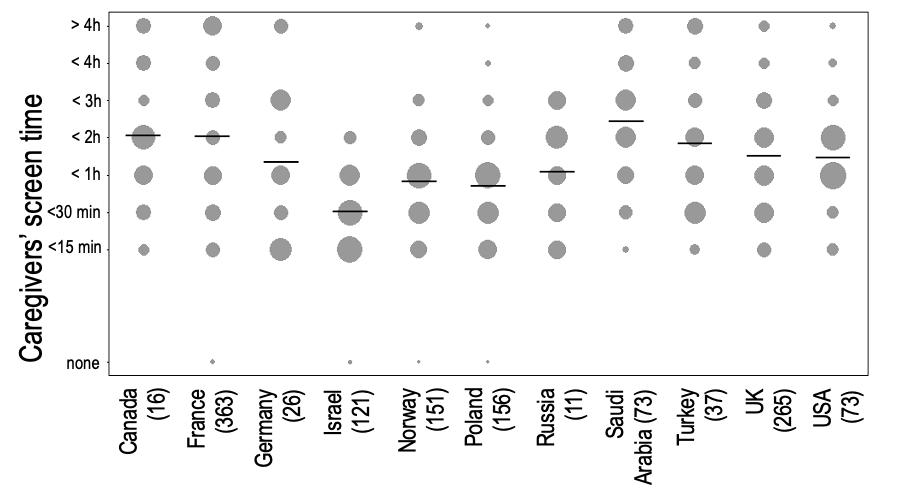


***Figure 4.*** Caregivers’ reports of their own screen time using data from the COVID-screen dataset. Note that data points that did not fall in the monotonous scale pre-registered for the current study (i.e., duration of screen time per week (1-2/w and 3-4/w) were excluded from the analysis. Circle size refers to proportions within a country, solid black lines indicate means to highlight trends in the data.

***
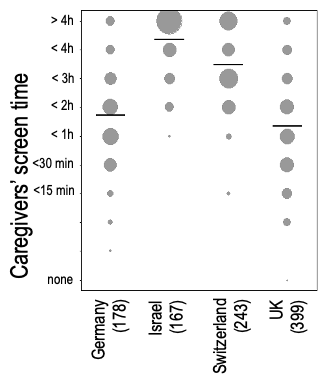
***

***Figure 5.*** Lockdown duration in the countries contributing to the COVID-language dataset and (B) COVID-screen dataset. Note that this was calculated based on the number of days between the date on which the T2 questionnaire was filled (the end of lockdown for that family due to the child starting daycare again or the end of data collection if lockdown was not yet complete) and the date on which nurseries, preschools and daycares shut in that region or country. Circle size refers to proportions within a country, solid black lines indicate means to highlight trends in the data.


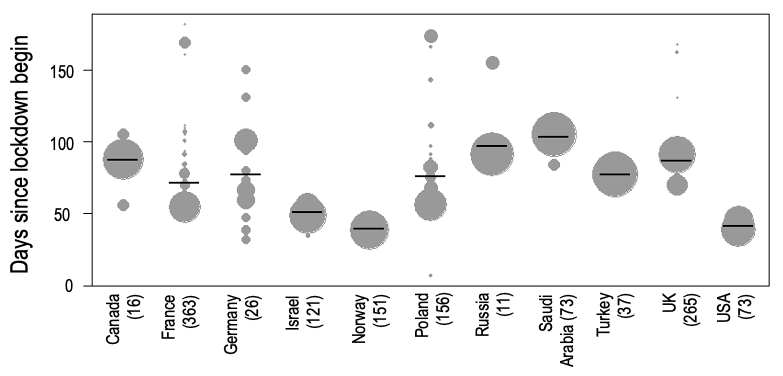


***Figure 6.*** Lockdown duration in the countries contributing to the COVID-screen dataset. Note that this was calculated based on the number of days between the date on which the T2 questionnaire was filled (the end of lockdown for that family due to the child starting daycare again or the end of data collection if lockdown was not yet complete) and the date on which nurseries, preschools and daycares shut in that region or country. Circle size refers to proportions within a country, solid black lines indicate means to highlight trends in the data.

**
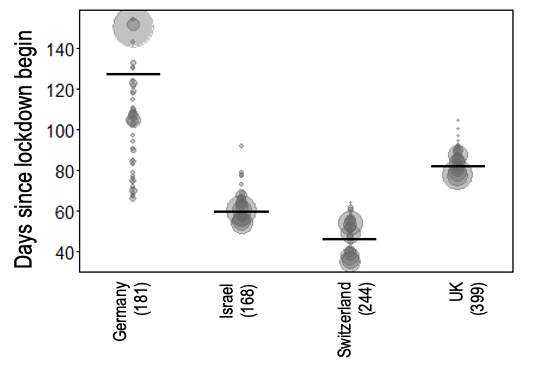
**

**Supplementary Information D:** Questions related to caregivers’ perception of potential positive and negative side-effects of children’s screen time

**Positive side-effects of screen time:** Does your child’s use of screens help you?

- No
- It frees me up time to work/ telecommute/ do household chores
- It frees up time for me
- This allows my child to have contact with family/friends
- It allows me to calm my child
- It allows me to create a bond with my child.

**Negative side-effects of screen time:** Does your child’s use of screens cause you difficulties?

- No
- I have trouble feeding my child
- My child has trouble falling asleep
- Screens lead to siblings fighting with one another
- My child is restless
- Screens lead to caregivers fighting with one another
- Screens make it difficult to interact with my child


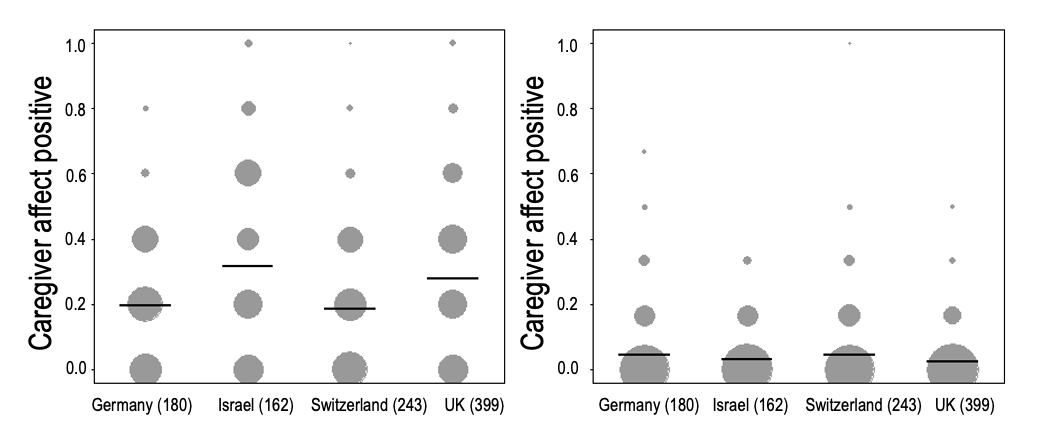


***Figure 7.*** Caregiver’s perception of positive (caregiver affect positive) and negative side-effects (caregiver affect negative) of children’s screen time in the COVID-screen dataset

**Supplementary Information E:** Assessment of whether the proportional odds assumption was fulfilled

Ordinal models make the assumption of proportional odds. In essence, this states that the effects of the predictors on the probability of the response to exceed a given value should be the same for all values of the response. We are not aware of the existence of an R package providing a function allowing for such a check for ordinal models in which random slopes effects are present. Hence, we checked for whether this assumption was fulfilled by fitting a series of binomial models for each of the ordinal models. The response variables for these models were the original response, dichotomized along each of the possible splits. For instance, with a response comprising the value 0, 1, 3, and 4, the response would be dichotomized three times as response ≧ 1, response ≧ 2, and response ≧ 3. We then fitted a model to each of the derived binary responses whereby the fixed and random effects model structure was identical to that of the original model. These models were fitted with binomial error structure and logit link function, using the function glmer of the package lme4 (version 1.1-27.1; Bates et al. 2015). To evaluate the results we plotted the estimated fixed effects coefficients, separately for each term, alongside those estimated by the original ordinal model.

In the case of **Model 1**, we found that the estimates did not vary much (Figure E1). For **Model 2**, we found that two of the estimates did not vary much (*age* and *caregiver.screentime*) but the others varied quite substantially (Figure E2). For **Model 2.SES** we found that four of the estimates did not vary much between the different binomial models. However, those of *lockdown.duration* and *lockdown.severity* and their interaction varied considerably, and that of *caregiver.affect* also varied to quite some extent (Figure E3). For **Model 3**, we found that more or less all estimates varied considerably between the different binomial models (Figure E4). However, these results must be treated very cautiously as in both cases several of the dichomizations of the response likely lead to complete separation problems (Field 2005), and several of the models also did not converge.


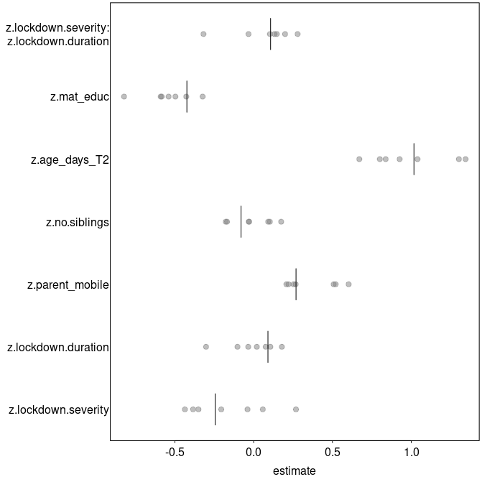


**Figure 8.** Fixed effects estimates in a series of binomial models with the ordinal response dichotomized at each possible splitting point (grey dots) and the original ordinal model (vertical line segments; **Model 1**). Labels on the Y-axis correspond to *SES* (z_matc_educ); *caregiver.affect* (z.affect.diff), *age* (z.age_days_T2), *siblings* (z.no.siblings), *caregiver.screentime* (z.parent_mobile).


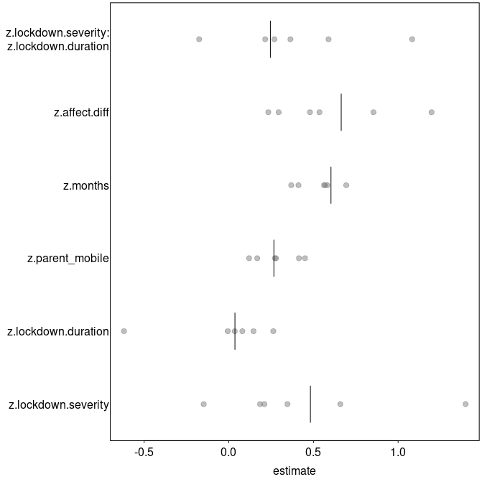


**Figure 9.** Fixed effects estimates in a series of binomial models with the ordinal response dichotomized at each possible splitting point (grey dots) and the original ordinal model (vertical line segments; Model 2). Labels on the Y-axis correspond to *caregiver.affect* (z.affect.diff), *age* (z.months), *caregiver.screentime* (z.parent_mobile).


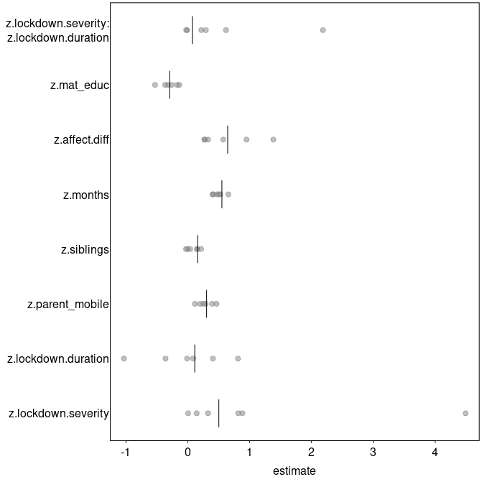


**Figure 10.** Fixed effects estimates in a series of binomial models with the ordinal response dichotomized at each possible splitting point (grey dots) and the original ordinal model (vertical line segments; **Model2.SES**). Labels on the Y-axis correspond to *SES* (z_matc_educ); *caregiver.affect* (z.affect.diff), *age* (z.months), *siblings* (z.siblings), *caregiver.screentime* (z.parent_mobile).


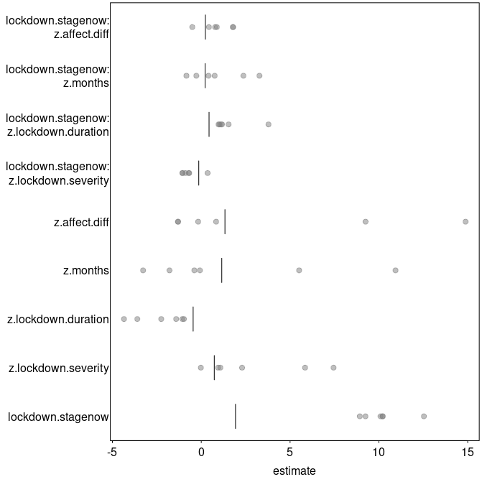


**Figure 11.** Fixed effects estimates in a series of binomial models with the ordinal response dichotomized at each possible splitting point (grey dots) and the original ordinal model (vertical line segments; **Model 3**). Labels on the Y-axis correspond to *caregiver.affect* (z.affect.diff), *age* (z.months), *siblings* (z.siblings), *caregiver.screentime* (z.parent_mobile).

**References:**

Bates, B., Maechler, M., Bolker, B. & Walker, S. (2015). Fitting Linear Mixed-Effects Models Using lme4. *Journal of Statistical Software*, 67, 1-48.

Field, A. (2005). *Discovering Statistics using SPSS*. Sage Publications. London.

**Supplementary Information F:**

The data sets used for the two models of vocabulary size comprised only 2 (receptive vocabulary size model) and 3 (expressive vocabulary size model) levels in the grouping factor *country*. As this likely leads to unreliable estimation of country level variation in the response, we fitted both models including *country* as fixed effect instead. Here we did not include interactions of the other fixed effects terms with *country*, as this would have hindered a comparison with the results of the original models.. In the case of both models, we found that the estimates changed very little when including *country* as a fixed rather than a random effect (compare Table F1 with Table B8 and Table F2 with Table B10).

**Table 13.** Results of the receptive vocabulary size model when including country as a fixed rather than a random effect.

| **term** | **Estimate** | **SE** |
| --- | --- | --- |
| (Intercept) | 0.622 | 0.402 |
| lockdown.stage | 0.324 | 0.092 |
| screen.time | 0.003 | 0.112 |
| caregiver.affect | 0.037 | 0.114 |
| age | 0.056 | 0.193 |
| SES | -0.010 | 0.102 |
| CountryIsrael | -0.865 | 0.506 |
| CountryUK | -0.376 | 0.492 |
| lockdown.stage:screen.time | -0.192 | 0.100 |
| lockdown.stage:caregiver.affect | -0.092 | 0.083 |
| lockdown.stage:age | -0.026 | 0.086 |

**Table 14.** Results of the expressive vocabulary size model when including country as a fixed rather than a random effect.

| **term** | **Estimate** | **SE** |
| --- | --- | --- |
| (Intercept) | 0.240 | 0.116 |
| lockdown.stage | 0.321 | 0.091 |
| screen.time | 0.023 | 0.111 |
| caregiver.affect | 0.061 | 0.113 |
| age | 0.213 | 0.115 |
| SES | -0.032 | 0.103 |
| lockdown.stage:screen.time | -0.191 | 0.100 |
| lockdown.stage:caregiver.affect | -0.096 | 0.083 |
| lockdown.stage:age | -0.036 | 0.085 |

**Supplementary Information G:**

Contribution (estimated standard deviation in link space) of the grouping factors in all full models. The columns in all tables denote the grouping factor *(grp)*, the effect *(effect)* where *(Intercept)* refers to a random intercept and all others to a random slope, the estimated standard deviation *(sd)* and its range (*min* and *max*), obtained when removing the levels of the grouping factor one at a time.

**Table 15. Model 1:**

| **grp** | **effect** | **sd** | **min** | **max** |
| --- | --- | --- | --- | --- |
| country | (Intercept) | 0.819 | 0.517 | 0.879 |
| country | caregiver.screentime | <0.001 | <0.001 | <0.001 |
| country | siblings | <0.001 | <0.001 | 0.094 |
| country | age | 0.211 | 0.154 | 0.240 |
| country | SES | 0.206 | 0.104 | 0.235 |
| country | lockdown.duration | 0.000 | <0.001 | 0.000 |

All covariates were z-transformed to a mean of zero and a standard deviation (sd) of one. Mean (sd) of the original variables were *lockdown.duratio*n: 70.359 (29.027); *caregiver.screentime*: 5.399 (1.83); *siblings*: 0.259 (0.61); *age*: 671.127 (213.289); *SES*: 4.588 (0.886)

**Table 16. Model 2:**

| **grp** | **effect** | **sd** | **min** | **max** |
| --- | --- | --- | --- | --- |
| country | (Intercept) | 0.643 | <0.001 | 0.809 |
| country | caregiver.screentime | <0.001 | <0.001 | <0.001 |
| country | age | 0.145 | <0.001 | 0.197 |
| country | caregiver.affect | 0.187 | <0.001 | 0.252 |

All covariates were z-transformed to a mean of zero and a standard deviation (sd) of one. Mean (sd) of the original variables were *caregiver.screentime*: 6.55 (1.854); *age*: 22.22 (6.428); *caregiver.affect*: 0.215 (0.233). Model stability could not be estimated for the estimated standard deviations since the data set comprised only three countries

**Table 17. Model 2.SES:**

| **grp** | **effect** | **sd** | **min** | **max** |
| --- | --- | --- | --- | --- |
| country | (Intercept) | 0.704 |  |  |
| country | caregiver.screentime | <0.001 |  |  |
| country | age | 0.174 |  |  |
| country | siblings | <0.001 |  |  |
| country | caregiver.affect | <0.001 |  |  |
| country | SES | <0.001 |  |  |

All covariates were z-transformed to a mean of zero and a standard deviation (sd) of one. Mean (sd) of the original variables were *caregiver.screentime*: 6.42 (1.903); *siblings*: 0.646 (0.72); *age*: 21.677 (6.533); *caregiver.affect*: 0.242 (0.244); *SES*: 4.365 (1.039)

**Table 18. Model 3:**

| **grp** | **effect** | **sd** | **min** | **max** |
| --- | --- | --- | --- | --- |
| newsubid | (Intercept) | 3.103 | 2.991 | 3.607 |
| country | I(lockdown.stage.code * caregiver.affect) | 0.180 | <0.001 | 0.275 |
| country | I(lockdown.stage.code * age) | <0.001 | <0.001 | 0.341 |
| country | caregiver.affect | 0.386 | <0.001 | 0.483 |
| country | age | 0.253 | <0.001 | 0.417 |
| country | lockdown.stage.code | 0.582 | <0.001 | 0.655 |
| country | (Intercept) | 0.643 | <0.001 | 1.103 |

All covariates were z-transformed to a mean of zero and a standard deviation (sd) of one. Mean (sd) of the original variables were *age*: 22.211 (6.426); *caregiver.affect*: 0.214 (0.233). *lockdown.stage* was dummy coded with pre-lockdown being the reference category and then centered to a mean of zero.

**Table 19. Model 4a:**

| **grp** | **effect** | **sd** | **min** | **max** |
| --- | --- | --- | --- | --- |
| country | (Intercept) | 0.463 |  |  |
| country.1 | lockdown.stage.code | <0.001 |  |  |
| country.2 | screen.time | <0.001 |  |  |
| country.3 | SES | <0.001 |  |  |
| country.4 | age | <0.001 |  |  |
| country.5 | caregiver.affect | <0.001 |  |  |
| country.6 | I(lockdown.stage.code * screen.time) | <0.001 |  |  |
| country.7 | I(lockdown.stage.code * age) | <0.001 |  |  |
| country.8 | I(lockdown.stage.code * caregiver.affect) | <0.001 |  |  |
| newsubid | (Intercept) | 1.377 | 1.289 | 1.610 |

All covariates were z-transformed to a mean of zero and a standard deviation (sd) of one. Mean (sd) of the original variables were *screen.time*: 1.59 (1.686); *age*: 16.44 (1.675); *caregiver.affect*: 0.238 (0.219); *SES*: 4.368 (0.875). *country* not shown because there were only two countries in the model.

**Table 20. Model 4b:**

| **grp** | **effect** | **sd** | **min** | **max** |
| --- | --- | --- | --- | --- |
| country | (Intercept) | 0.463 | <0.001 | 0.001 |
| country.1 | lockdown.stage.code | <0.001 | <0.001 | 0.054 |
| country.2 | screen.time | <0.001 | <0.001 | <0.001 |
| country.3 | SES | <0.001 | <0.001 | <0.001 |
| country.4 | age | <0.001 | <0.001 | <0.001 |
| country.5 | caregiver.affect | <0.001 | <0.001 | <0.001 |
| country.6 | I(lockdown.stage.code * age) | <0.001 | <0.001 | <0.001 |
| country.7 | I(lockdown.stage.code * screen.time) | <0.001 | <0.001 | <0.001 |
| country.8 | I(lockdown.stage.code * caregiver.affect) | <0.001 | <0.001 | 0.089 |
| newsubid | (Intercept) | 1.228 | 1.135 | 1.535 |

All covariates were z-transformed to a mean of zero and a standard deviation (sd) of one. Mean (sd) of the original variables were *screen.time*: 1.59 (1.686); *age*: 16.44 (1.675); *caregiver.affect*: 0.238 (0.219); *SES*: 4.368 (0.875).
